# Genome-wide analyses implicate 33 loci in heritable dog osteosarcoma, including regulatory variants near *CDKN2A/B*

**DOI:** 10.1186/gb-2013-14-12-r132

**Published:** 2013-12-12

**Authors:** Elinor K Karlsson, Snaevar Sigurdsson, Emma Ivansson, Rachael Thomas, Ingegerd Elvers, Jason Wright, Cedric Howald, Noriko Tonomura, Michele Perloski, Ross Swofford, Tara Biagi, Sarah Fryc, Nathan Anderson, Celine Courtay-Cahen, Lisa Youell, Sally L Ricketts, Sarah Mandlebaum, Patricio Rivera, Henrik von Euler, William C Kisseberth, Cheryl A London, Eric S Lander, Guillermo Couto, Kenine Comstock, Mike P Starkey, Jaime F Modiano, Matthew Breen, Kerstin Lindblad-Toh

**Affiliations:** 1 Broad Institute of MIT and Harvard, Cambridge, MA, USA; 2 FAS Center for Systems Biology, Harvard University, Cambridge, MA, USA; 3 Science for Life Laboratory, Department of Medical Biochemistry and Microbiology, Uppsala University, Uppsala, Sweden; 4 Department of Molecular Biomedical Sciences, College of Veterinary Medicine & Center for Comparative Medicine and Translational Research, North Carolina State University, Raleigh, NC, USA; 5 Department of Clinical Sciences, Cummings School of Veterinary Medicine at Tufts University, North Grafton, MA, USA; 6 Oncology Research Group, Animal Health Trust, Newmarket, Suffolk, UK; 7 Canine Genetics Research Group, Animal Health Trust, Newmarket, Suffolk, UK; 8 Department of Urology, University of Michigan, Ann Arbor, MI, USA; 9 Department of Animal Breeding and Genetics, Uppsala University, Uppsala, Sweden; 10 Department of Clinical Sciences, Swedish University of Agricultural Sciences, Uppsala, Sweden; 11 Department of Veterinary Clinical Sciences and Veterinary Medical Center, The Ohio State University, Columbus, OH, USA; 12 Department of Veterinary Biosciences, College of Veterinary Medicine, The Ohio State University, Columbus, OH, USA; 13 Couto Veterinary Consultants, Hilliard, OH, USA; 14 Masonic Cancer Center, University of Minnesota, Minneapolis, MN, USA; 15 Department of Veterinary Clinical Sciences, College of Veterinary Medicine, University of Minnesota, Saint Paul, MN, USA; 16 Cancer Genetics Program, UNC Lineberger Comprehensive Cancer Center, Chapel Hill, NC, USA

## Abstract

**Background:**

Canine osteosarcoma is clinically nearly identical to the human disease, but is common and highly heritable, making genetic dissection feasible.

**Results:**

Through genome-wide association analyses in three breeds (greyhounds, Rottweilers, and Irish wolfhounds), we identify 33 inherited risk loci explaining 55% to 85% of phenotype variance in each breed. The greyhound locus exhibiting the strongest association, located 150 kilobases upstream of the genes *CDKN2A/B*, is also the most rearranged locus in canine osteosarcoma tumors. The top germline candidate variant is found at a >90% frequency in Rottweilers and Irish wolfhounds, and alters an evolutionarily constrained element that we show has strong enhancer activity in human osteosarcoma cells. In all three breeds, osteosarcoma-associated loci and regions of reduced heterozygosity are enriched for genes in pathways connected to bone differentiation and growth. Several pathways, including one of genes regulated by *miR124*, are also enriched for somatic copy-number changes in tumors.

**Conclusions:**

Mapping a complex cancer in multiple dog breeds reveals a polygenic spectrum of germline risk factors pointing to specific pathways as drivers of disease.

## Background

Osteosarcoma (OS) is an aggressive cancer characterized by early metastasis, primary onset in children and adolescents, and high mortality rates (30% to 40%) [1]. Recent work suggests that OS arises when osteoblast differentiation from mesenchymal precursors is disrupted by genetic or epigenetic factors [2]. While no structural variants specific to OS have been identified, somatic alterations in tumors are common and often affect suppressor genes *RB1*, *TP53*, and the CDK4 inhibitors *CDKN2A/B*[2]. Germline mutations in *RB1* and *p53*, two genes essential for OS development, can increase disease risk [2-4]. The only genome-wide association study (GWAS) of OS in humans found two significant associations, one genic (*GRM4*) and the other in a large gene desert, suggesting inherited variation in regulatory elements underlies susceptibility [5].

OS in dogs is a spontaneously occurring disease with a global tumor gene expression signature indistinguishable from human pediatric tumors [6,7] and, while relative age of onset is higher in dogs, their clinical progression is remarkably similar [8]. Both human and canine OS most commonly arise at the ends of the long bones of the limbs and metastasize readily, usually to the lungs [9]. Unlike human OS, canine OS is a highly heritable disease with some large and giant dog breeds at >10× increased risk, notably greyhounds (mortality from OS = 26%), Rottweilers (17%), and Irish wolfhounds (IWH, 21%) [10-12]. While size and hormonal factors influence risk, variable rates among the larger size breeds suggest breed-specific risk factors [13]. The greyhound American Kennel Club (AKC) registered dogs, a subpopulation that tends to be taller than the racing dogs, has very low rates of OS (G. Couto, unpublished observations).

Mapping disease genes using GWAS in dog breeds, each effectively a genetic isolate only a few hundred years old, requires approximately 10× fewer markers and samples than in human populations [14]. However, population structure, cryptic relatedness, and extensive regions of near fixation in breeds complicate GWAS analysis and to date, few studies have successfully mapped risk factors for complex, multigenic diseases [15]. Here, we use new methods for analyzing breed populations to identify genomic loci associated with OS in the first multibreed association study of a highly polygenic canine disease. We explain the majority of the OS phenotype variance in three high-risk breeds, identify a common regulatory risk factor, and reveal novel genes and pathways underlying this poorly understood disease.

## Results

### Population genetics of GWAS dog breeds

We genotyped 334 greyhounds, 166 Rottweilers, and 174 IWH on the 170,000 SNP Illumina canine HD arrays, removing SNPs with call rates <95%, and one dog from each pair with the same phenotype and genetic relatedness >0.25, preferentially retaining younger cases and older controls. The final dataset (169,010 SNPs) included 267 racing greyhounds (153 affected (A) + 114 unaffected (U)), 135 Rottweilers (80 A + 55 U), 141 IWH (76 A + 65 U), and 19 AKC greyhounds. The ratio of females to males was approximately equal in cases and controls in the greyhounds (0.72 and 0.75 for A and U) and Rottweilers (1.11 and 1.04), and more skewed in IWH (1.62 and 1.41).

**Figure 1:**
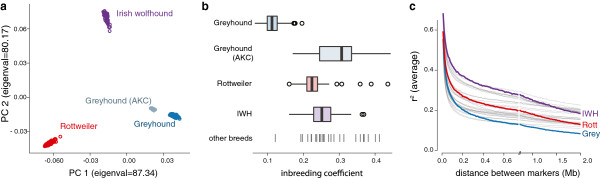
**The three GWAS breeds (high risk of OS) and the AKC greyhounds (low risk of OS) are genetically distinct and highly inbred populations. (a)** Along the top two principal components of variation, the three breeds (Rottweilers (*n* = 135, red), Irish wolfhounds (IWH, *n* = 141, purple), and racing greyhounds (*n* = 267, dark blue) ) are each tight clusters equidistant apart, with the AKC subpopulation of greyhounds (*n* = 19, light blue) near but not overlapping the racing dogs. **(b)** Estimate of the inbreeding coefficient (IC) shows the racing greyhounds as the least inbred, on average, while the other three populations fall within the range seen in an earlier survey of 28 dog breeds (grey bars mark average IC in breed; two outliers at 0.53 and 0.62 not shown) [17]. **(c)** The extent of linkage disequilibrium, measured as average pair-wise r^2^, drops below 0.2 at 196 kb, 632 kb, and 1,533 kb in the greyhounds, Rottweilers, and IWH, respectively.

The three breeds are visibly discrete genetic populations; the AKC greyhounds cluster near but distinct from their racing brethren (Figure 1a). The racing greyhound population is the least inbred, likely reflecting a large effective population size, (inbreeding coefficient θ = 0.11 +/− 0.02), followed by the Rottweilers (θ = 0.23 +/− 0.04), IWHs (θ = 0.25 +/− 0.04), and AKC greyhounds (0.30 +/− 0.07) (Figure 1b) [16]. While linkage disequilibrium in all breeds is long, as compared to human populations, it varies markedly by breed, with average r^2^ dropping below 0.2 at 196 kb in the greyhounds, 632 kb in the Rottweilers, and 1,533 kb in the IWH, suggesting GWAS regions will be shortest in greyhound, facilitating identification of associated functional elements in this breed (Figure 1c).

### GWAS identifies 33 regions associated with osteosarcoma

**Figure 2:**
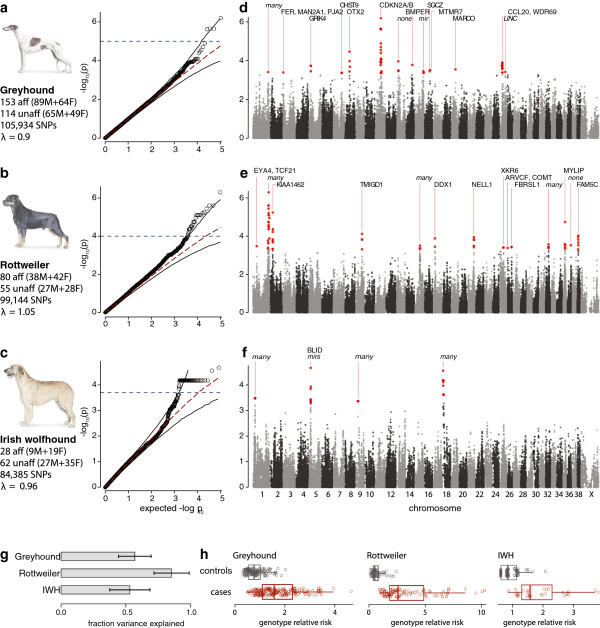
**Mixed-model GWAS corrects for population structure and identifies 33 OSA associated loci explaining a large fraction of phenotype variance.** In each breed, the QQ plots show no evidence of stratification relative to the expected distribution, identifying nominal significance at -log_10_p of 3.5 and the 95% empirically determined confidence intervals (dashed grey line) at -log_10_p of **(a)** 5 in the greyhounds, **(b)** 4 in the Rottweilers, and **(c)** 3.7 in IWH. In the IWH, a plateau of SNPs at *P* = 6.6 × 10^-5^ corresponds to a 1.65 Mb haplotype on chromosome 18 peaking at the gene *GRB10*. **(d)** In greyhounds, 14 loci have *P*<0.0005, with one locus, on chromosome 11, exceeding 95% confidence intervals (dashed lines). **(e)** In Rottweilers, 15 and 6 loci are identified, **(f)** while only four and two loci are identified in IWHs. **(g)** For each breed, the phenotype variance explained by the associated loci, broadly defined by SNPs with r^2^ >0.2 within 5 Mb of the peak SNP, exceeds 50%. In greyhounds, the 14 regions explain 56.9% +/− 12.5%, in Rottweilers, 15 regions explain 85.3 +/− 13.6%, and in IWH, four regions explain 53.1 +/− 15.5%. **(h)** For each affected dog (red circles) and unaffected dog (black circles), we estimated their relative risk based on the genotypes and odds ratio of the top SNP from each region for the breed (Table 1), showing that even using a small number of SNPs we see clear differences between the cases and the controls.

We tested for association between OS and germ-line variants with minor allele frequency >0.05 in each of the three breeds independently, controlling for cryptic relatedness and population structure using a mixed model approach with the top principal component as a covariate [18,19]. We identified all SNPs with either significant association (exceeding 95% confidence intervals defined empirically using 1,000 random phenotype permutations) or suggestive association (*P*<0.0005; Figure 2a-f; Methods) and defined regions of association using linkage disequilibrium (Table 1, Additional file 1: Figure S1). The proportion of very young cases in our IWH dataset allowed us to focus on the more powerful comparison of young cases and older controls (Additional file 1: Figure S2; 28 affected <6 years old and 62 unaffected >6 years old).

**Table 1:** Osteosarcoma associated loci identified by independent GWAS in three dog breeds with high rates of the disease

**SNP**	**chr**	**Position**	***P***	**Risk allele**	**OR**	**f (A)**	**f (U)**	**Region start-end**	**Size (kb)**	**Genes**
***Greyhound***										
BICF2P133066	11	44405676	6.4E-07	A	1.26	0.84	0.65	44392734-44414985	22	None
BICF2P1421479	8	35448126	3.4E-05	C	1.36	0.12	0.03	35433142-35454649	22	OTX2 (50 kb)
BICF2S23118341	13	14588716	1.1E-04	T	1.20	0.34	0.19	14549973-14645634	96	None
BICF2S23325120	25	21912859	1.3E-04	A	1.19	0.56	0.41	21831580-21921256	90	None
BICF2P66597	14	49193217	1.6E-04	G	1.19	0.37	0.23	48831824-49203827	372	BMPER
BICF2P1194727	5	16085937	1.8E-04	G	1.23	0.28	0.14	16071171-16152955	82	GRIK4
BICF2G63051809	19	34134931	2.8E-04	T	1.21	0.80	0.67	33963105-34145310	182	EN1, MARCO
BICF2G630813090	16	43669044	3.0E-04	C	1.16	0.64	0.48	43665149-43737129	72	MTMR7
BICF2G630418573	15	63780452	3.4E-04	A	1.26	0.91	0.81	63767963-63800415	32	None
TIGRP2P215623	16	40896559	3.5E-04	C	1.36	0.97	0.89	40883517-41081510	198	SGCZ
TIGRP2P331221	25	43485109	3.8E-04	G	1.23	0.22	0.11	43476429-43528145	52	CCL20
BICF2S23516022	1	112990983	3.8E-04	C	1.21	0.82	0.69	112977233-113081800	105	CD3EAP, ERCC1, ERCC2, FOSB, PPP1R13L
TIGRP2P45171	3	5564882	4.0E-04	T	1.20	0.79	0.68	5162058-6465753	1,304	FER, MAN2A1, PJA2
BICF2P1090686	7	64672328	4.2E-04	C	1.16	0.57	0.43	64631053-64703475	72	CHST9
***Rottweiler***										
BICF2P1115364	1	116524913	5.0E-07	G	1.32	0.71	0.39	115582915-116790630	1,208	Many (35 genes)
BICF2P411325	2	19515571	5.8E-06	C	1.43	0.91	0.73	19212450-19542015	330	KIAA1462
BICF2P1210630	1	122048812	1.1E-05	C	1.30	0.73	0.46	122033806-122051988	18	C19orf40, CEP89, RHPN2
TIGRP2P407733	35	18338700	1.8E-05	A	1.28	0.51	0.29	18326079-18345318	19	None
BICF2P341331	9	47659782	7.6E-05	A	1.28	0.53	0.28	47647012-47668054	21	BLMH, TMIGD1
BICF2P1129874	38	11714169	9.4E-05	T	1.24	0.49	0.26	11252518-11739329	487	FAM5C
TIGRP2P286750	21	46283811	1.1E-04	C	1.37	0.84	0.70	46231985-46363479	131	NELL1
BICF2S23533459	17	14472761	1.3E-04	C	1.39	0.18	0.01	14465884-14482152	16	None
BICF2G630590368	32	25147661	2.7E-04	A	1.35	0.95	0.81	25136302-25156153	20	EMCN
BICF2P92014	36	29651125	3.0E-04	A	1.22	0.66	0.42	29637804-29663408	26	None
TIGRP2P200071	15	38987072	3.1E-04	T	1.27	0.82	0.59	37986345-39974762	1,988	Many (15 genes)
BICF2P1164085	1	29775073	3.3E-04	G	1.24	0.46	0.23	29405587-29914411	509	EYA4, TCF21
BICF2S23712115	26	32385934	3.7E-04	G	1.35	0.91	0.76	32374093-32428448	54	ARVCF, C22orf25, COMT
BICF2G63095567	25	29671618	3.9E-04	G	1.30	0.23	0.05	29658978-29767164	108	XKR6
BICF2P841536	26	3537143	4.2E-04	A	1.26	0.48	0.30	3529343-3550075	21	FBRSL1
***Irish Wolfhound***										
BICF2S23746532	5	15264066	2.1E-05	A	1.40	0.45	0.16	14720254-15466603	746	BLID
BICF2P1466354	18	4937944	2.7E-05	C	1.36	0.56	0.21	4266743-5854451	1,588	C7orf72, COBL, DDC, FIGNL1, GRB10, IKZF1, VWC2, ZPBP
BICF2P1225386	1	17742179	3.2E-04	C	1.31	0.46	0.19	16768869-18150476	1,382	BCL2, KIAA1468, PHLPP1, PIGN, RNF152, TNFRSF11A, ZCCHC2
BICF2P1125643	9	19623231	4.3E-04	C	1.75	0.14	0.02	18896060-19633155	737	ABCA5, KCNJ16, KCNJ2, MAP2K6

OR is the conditional odds ratio estimated from beta values calculated by EMMAX.

Within each breed, associated regions (*P*<0.0005) explain the majority of the phenotype variance: 57% in the greyhound (14 loci), 53% in the IWH (4 loci), and 85% in the Rottweilers (15 loci) (Figure 2g, Additional file 1: Figure S3). The overall genotype relative risk estimated for each dog, based on the risk contributed by each locus, shows a clear separation between cases and controls in each breed (Figure 2h), even at more stringent significance thresholds (Additional file 1: Figure S4). None of the top scoring SNPs was strongly correlated with sex in a genome-wide analysis (Additional file 1: Table S1), and including sex as a covariate revealed no new regions of strong association. Although earlier work on monogenic traits in dogs suggested risk factors often are shared between breeds [20], we see no overlap in regions of association between the breeds and a meta-analysis of the three breeds yielded no significant associations (Additional file 1: Figure S5). Three possible explanations for this observation are: (1) cancer risk factors are abundant in the overall dog population and each breed inherited different subsets; (2) we cannot detect the shared associations in the other breeds at our current sample sizes; and (3) the risk haplotypes are fixed or nearly fixed in the other breeds, and thus undetectable by association.

**Figure 3:**
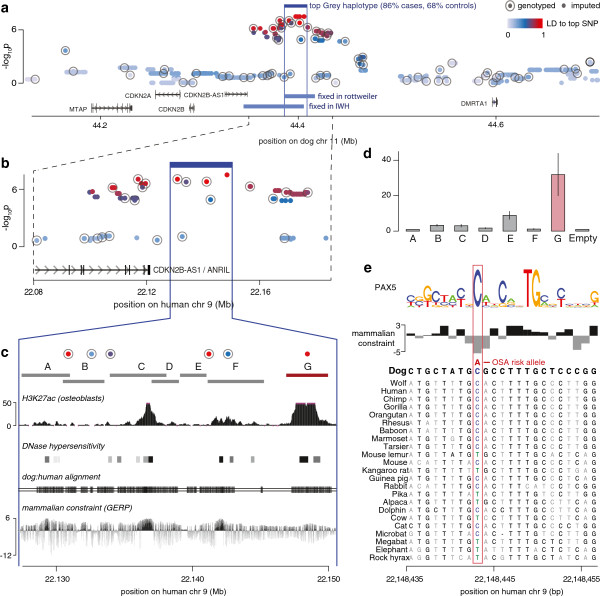
**Identification of the top associated variant and functional analysis on chromosome 11. (a)** We targeted 2.5 Mb around the greyhound GWAS peak on chromosome 11 for dense sequencing (15 dogs) and finemapping (180 cases and 115 controls). Imputation and association testing of sequenced variants narrowed the peak of association in greyhounds dramatically to a 15 kb risk haplotype (chr11:44390633–44406002), telomeric of the genes *CDKN2A* and *CDKN2B*, that is nearly fixed in both the Rottweilers (98% in cases and 96% in controls) and IWH (95% in cases and 92% in controls). **(b)** The top haplotype (blue vertical lines) maps to a locus downstream of the non-coding gene *ANRIL* on human chromosome 9 (hg19). **(c)** We tiled the human chromosome 9 region with luciferase assays and assayed the function in osteosarcoma cell lines compared to renilla. Potential markers of function in the region include H3K27 acetylation in osteoblasts and DNase hypersensitivity clusters (assayed from 125 cell types), most notably in regions that align between the dog and human genomes in a Multiz alignment of 46 species and are constrained across mammals as measured by Genomic Evolutionary Rate Profiling (GERP) [21-24]. **(d)** Of the seven non-control luciferase assays, four (B, C, E, and G) showed a significant increase compared to empty vector. Construct G showed by far the strongest increase with an approximate 32-fold increased activity suggesting a strong enhancer. **(e)** This fragment contains one of the top SNPs (Canfam2.0 chr11:44405676) which has a constrained reference allele C corresponding to a predicted transcription factor binding site, while the OS associated allele, A, is not found among 29 mammals or the wolf.

We tested the top 33 GWAS regions for fixation of the risk allele (frequency >0.95) in the other two breeds. In eight regions, the risk haplotype is fixed in one of the other two breeds, but in only one is it fixed in both: the risk allele tagging the top greyhound locus (A=0.84,U=0.65), on chromosome 11, is fixed in both Rottweilers (0.97) and IWH (0.95) (Table 1). This haplotype occurs at lower frequency in the AKC greyhounds (0.61) and in a panel of 28 other breeds from a previously published dataset (0.51% +/− 0.24) [17]. We sequenced the locus (chr11:43.0-48.9 Mb ) in eight greyhound cases and seven controls, and densely genotyped and imputed 180 greyhound cases and 115 controls (Figure 3a). This narrowed the region of association to a 15 kb non-coding region (chr11:44,390,633-44,406,002) near *CDKN2A* (encodes p16^INK4a^ and p14^ARF^), *CDKN2B* (p15^INK4b^), and the antisense non-coding gene *CDKN2B-AS1/ANRIL* (Figure 3a). Both *CDKN2A* and *CDKN2B* function as cell cycle regulators and tumor suppressors.

The risk haplotype at 11q16 is syntenic to a non-coding regulatory region on human chromosome 9p21 (Figure 3b), and the alignment between dog and human includes DNase hypersensitivity sites and peaks of H3K27 acetylation characteristic of active enhancers (Figure 3c) [25,26]. The human 9p21 locus is deleted in 5% to 21% of human OS [27], the absence of p16INK4a expression is correlated with decreased survival in pediatric OS patients [28], and increased p16 expression is predictive of better response to chemotherapy [29]. Thus, we hypothesized that the variant(s) carried on the risk haplotype disrupts enhancer elements upstream of the *CDKN2A/B* locus, thereby altering expression of one or more genes in the region.

We assayed the risk haplotype for regions of enhancer activity using renilla/firefly luciferase assays in the human OS U2OS cell line, tiling the region with seven fragments that were PCR-amplified from human genomic DNA (Figure 3c). Several fragments showed enhancer activity, and one increased luciferase expression >30-fold (Figure 3d). The only greyhound variant identified in this fragment region, a SNP, is also the top associated variant in the greyhound finemapping/imputation dataset (dog chr11:44405676; human chr9:22,148,443, *P* = 3 × 10^-8^) (Figure 3a,b) (Additional file 1: Table S2). Additional genotyping validated the imputation (186 dogs genotyped; 98.9% concordance). While this position is strongly constrained to a C or T across mammals, including wolves [30], the OS risk allele, A, is found in 87% of greyhound cases and 68% of greyhounds controls (Figure 3e). We analyzed the locus with two different transcription factor binding motif analysis tools, FIMO [31] and TOMTOM [32]. Just one motif, for PAX5, was significantly detected by both tools and was specific to the non-risk allele (C), suggesting that the risk allele (A) will disrupt binding (Figure 3f, Additional file 1: Figure S6). PAX5 regulates both B-cell and osteoblast differentiation and bone formation [33,34].

We tested for association of chr11:44405676 to osteosarcoma in eight more breeds with high rates of OS. We found the greyhound risk allele at very high frequency in the two other GWAS breeds, Rottweilers (92A + 77U, F_A_ = 0.98, F_U_ = 0.97) and IWH (27A + 31U, F_A_ = 0.93, F_U_ = 0.92), with the risk allele slightly more common in cases, and correlated with OS in Leonbergers (30A + 25U, F_A_ = 0.77, F_U_ = 0.62, *P* = 0.09) and great Pyrenees (16A + 21U, F_A_ = 0.78, F_U_ = 0.62, *P* = 0.14). Analyzed together, these four breeds replicate the association seen in the greyhounds (p_CMH_ = 0.03, p_CMH_ = 2 × 10^-8^ with greyhounds included) and no odds-ratio heterogeneity was seen between breeds (p_Breslow-Day_ = 0.51) (Additional file 1: Table S3) [35]. We found no association with OS risk in mastiffs, Labrador retrievers, great Danes, and golden retrievers. Although small samples limit statistical power, these results suggest the effect of the risk variant may depend on the genetic background in each breed. Pooled sequencing data from an earlier project [30] show that the OS risk allele (A) is never seen in wolves but is common in purebred dogs (approximately 50%). The syntenic human locus at 9p21 has one of the densest known concentrations of regulatory elements in the human genome [36]. Breed-specific variation in nearby functional elements may modulate the effect of the chr11:44405676 variant.

### Pathway analysis of osteosarcoma associated regions

**Figure 4:**
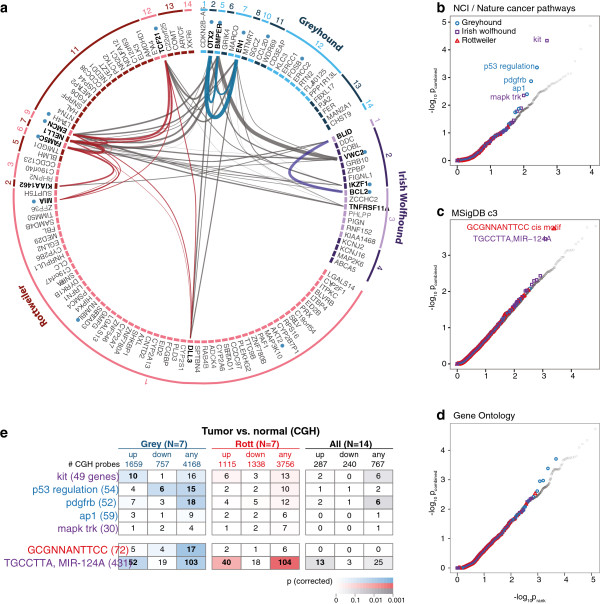
**Connectivity and enrichment analysis identifies pathways linked to OS in multiple breeds. (a)** Most of the associated regions in each breed contain one or more genes (black text). Genic regions are shown for the greyhounds (blue), IWH (purple), and Rottweilers (red), with hue alternating between light and dark to distinguish regions, numbered as in Table 1. GRAIL [37] analysis identified non-random connectivity (*P*<0.05) between associated genes (bold text), both within breeds (blue, purple, and red arched lines for greyhounds, IWH, and Rottweilers) and between breeds (grey arched lines). Twelve regions contain genes (blue dots) connected to the key word ‘bone’, one of the top terms identified by GRAIL (Table S4). **(b, c, d)** When gene set enrichment *P* values for the associated regions and regions of reduced variability are combined, most sets shows no inflation compared to background (grey circles; RRV *P* values from 28 other breeds). However a small number are inflated, including **(b)** five from the NCI pathway interaction database [47], **(c)** two from the Molecular Signatures database of shared cis-regulatory motif based sets [48], and **(d)** 0 from the Gene Ontology database. **(e)** Of the seven gene sets (colors match discovery breed), five are significantly enriched (bold numbers) in regions that are aberrant in all greyhound (blue), all Rottweiler (red), or all dogs (black) for which we compared normal and tumor DNA using comparative genomic hybridization. Boxed numbers show number of genes in gene set overlapping CGH regions; ‘up’ and ‘down’ indicates gain or loss in all samples; ‘any’ indicates all samples are either amplified or deleted.

While the breeds do not segregate for the same risk variants, their shared predisposition to OS suggests the risk alleles may disrupt the same cellular pathways. All but four of the 33 OS associated regions contain known genes. The majority have just one gene (60%), but a handful have considerably more, including the top Rottweiler region (35 genes) and three out of four IWH regions (4, 7, and 8 genes) (Table 1). We analyzed the GWAS regions using GRAIL, a statistically robust, web-based method that uses published scientific abstracts to find gene relationships connecting distinct genomic regions [37]. We identified significant connectivity between OS associated regions both within and between breeds through key terms including ‘bone’ (13 loci), ‘differentiation’ (13 loci), and ‘development’ (9 loci) (Figure 4a, Additional file 1: Table S4). For example, *OTX2*, an oncogenic orthodenticle homeobox protein that directly activates cell cycle genes and inhibits differentiation in medulloblastomas [38], is strongly associated with OS in the greyhounds. GRAIL connects *OTX2* with genes in six other risk loci (*P*<0.05): two negative regulators of osteoblast differentiation *BMPER* and *VWC2*[39]; *EN1*, a modulator of osteoblast differentiation and proliferation [40]; *DLL3*, a notch ligand implicated in human skeletal growth disorders [41]; *TCF21*, a tumor suppressor that regulates mesenchymal-epithelial cell transitions; and *EMCN*, a mucin-like anti-adhesion membrane protein and hematopoietic stem cell marker [42]. In a second network, GRAIL connects three genes that regulate bone formation - the osteoblast differentiation enhancer *FAM5C*[43]; *NELL1*, a regulator of osteoblast differentiation and ossification [44]; and *TNFRSF11A*, an essential mediator of osteoclast development [45] - and the pro-apoptotic gene *BLID*, frequently deleted in human breast, lung, ovarian, and cervical cancers [46].

### Fixed and selected loci in breeds contribute to disease risk

It is likely that alleles that are fixed or at high frequency in breeds, and thus undetectable by GWAS, contribute to OS risk, as seen at the CDKNA2/B locus. In each breed a substantial portion of the genome is comprised of fixed regions (minor allele frequency <0.05) longer than 250 kb: 2.8% of the autosomal genome in the greyhounds, 2.9% in the Rottweilers, and 7.6% in IWH (Additional file 1: Figure S7a; 25.9%, 30.5%, and 31.5% for chromosome X). These fixed regions overlap genes involved in bone development and OS, including *RB1* (IWH), *FOS* (Rottweilers), *RUNX2* (Rottweilers), *CCNB1* (IWH), *COL11A2* (greyhounds), and *POSTN* (IWH and greyhounds) [49]. We tested genomic regions fixed in all three breeds for gene set enrichment using INRICH, empirically measuring significance through 100,000 permutations matched for region size, SNP density, and gene number [50]. Among eight sets of microRNAs implicated in OS pathobiology [51-55], we found significant enrichment for one associated with pathogenesis and progression of OS (5/27 genes, *P* = 0.017, *P*_corrected_ = 0.041, Additional file 1: Table S6) [51].

We next analyzed the fraction of the genome that shows exceptionally low variation in OS breeds compared to 28 other breeds, including regions of incomplete fixation. We defined these regions of reduced relative variability (RRVs) by comparing each OS breed to up to 28 other dog breeds and focusing on the 1% least variable 150 kb regions [17]. RRVs totaled 2.9% (277 regions), 2.9% (344 regions), and 3.1% (387 regions) of the genome in the greyhounds, Rottweilers, and IWH, respectively (Additional file 1: Figure S7b). We tested the RRVs for gene set enrichment using INRICH and combined these *P* values with those from a matched analysis of the GWAS regions. We hypothesized that, if genes in RRVs contribute to OS risk, we should see the same gene pathways enriched in the two analyses. While, as expected, the vast majority of gene sets in the studied breeds showed no increase in significance compared to the background distribution, seven gene sets are markedly inflated. This includes three pathways (KIT, p53, and PDGFRB) in a list curated by the National Cancer Institute and Nature Publishing Group (Figure 4b) and, from the Molecular Signatures Database, two gene sets defined by cis-regulatory motifs - targets of MIR - 124A (TGCCTTA) and a highly conserved motif with no known transcription factor match (Figure 4c) [47]. We found only weak *P* value inflation in the Gene Ontology analysis (Figure 4d).

### GWAS pathways enriched for somatic mutations in OS tumors

Our analysis of the OS breeds demonstrates that inherited variants are major factors for determining whether a dog develops OS. As somatic changes in the tumor also contribute to progression of the disease, we hypothesize that genes affected by these changes will be enriched in the same pathways as the inherited variants. We investigated the frequency and distribution of somatic DNA copy number aberrations (CNAs) in 22 OS tumor samples (12 greyhounds, 10 Rottweilers) using 26 kb-resolution genome-wide array-based comparative genomic hybridization analysis (array-CGH).

While the CGH profiles exhibit the extensive karyotypic instability characteristic of OS [56], they are remarkably conserved between the two breeds, with no significant regional differences in DNA copy number status (defined as corrected *P*<0.05; Additional file 1: Figure S8a,b,c). Moreover, the genome-wide CGH profiles of dog and human OS are broadly consistent, both in the frequency and relative distribution of CNAs (Additional file 1: Figure S8d), including genes associated with OS pathogenesis [56], such as *MYC* gain (60% in dog/67% in human), *RB1* loss (36%/33%), *RUNX2* gain (45%/67%), and *CDKN2A/B* loss (73%/67%).

Using the GISTIC algorithm [57], we identified discrete regions that had statistically high CNA frequency in canine tumors relative to the globally chaotic genomic background of OS, suggestive of specific gene targets strongly associated with disease pathogenesis. The most significant was a 2.5 Mb region at 11q16 (chr11:43,615,205-46,137,412) encompassing the *CDKN2A* and *CDKN2B* genes, with the strongest signal (*P*_corrected_ = 1.5 × 10^-12^) at chr11:44,304,860-44,308,340 between *CDKN2B* and *CDKN2A-AS1*, approximately 100 kb from the top greyhound GWAS SNP*.* This region was deleted in 73% of tumors (9/12 greyhounds, 7/10 Rottweilers), of which 59% were consistent with homozygous deletion (7/12 greyhounds, 6/10 Rottweilers). No other regions identified by GISTIC overlapped GWAS loci, reinforcing the fundamental role of the *CDKN2A/B* region in disease pathogenesis.

We tested the subset of the CGH samples (7 greyhounds and 7 Rottweilers) also included in the GWAS analysis and found that five of the top 29 GWAS SNPs are moderately associated with tumor gain or loss, most significantly at the gene *BLMH*, a candidate tumor suppressor gene for hepatocellular carcinoma [58] (Additional file 1: Table S5). Furthermore, certain probes were enriched for genomic imbalance; the fraction of probes gained or lost in all Rottweilers (*n* = 8,087, 4.95%), all greyhounds (*n* = 8,781, 5.35%), or all 14 dogs (*n* = 1,603, 0.98%) was much higher than expected by random chance (*P*_binomial_ = 2.71%, 1.3%, and 0.04%, respectively). We found putative human OS driver genes deleted in human tumors [59] are among those lost in all greyhounds (*ARHGAP22*, *ARID5B*, *RCBTB1*; INRICH enrichment *P* = 0.0004), all Rottweilers (*LHFP*; *P* = 0.13), and all dogs (*AIFM2*, *TSC22D1*; *P* = 0.024).

Of the seven gene sets enriched in the GWAS + RRV analysis, five are also enriched in the CGH regions in one or both breeds (Figure 4e). In particular, genes with a MIR-124A cis-regulatory motif, identified in IWH, showed significant enrichment in both Rottweiler and greyhound tumors. MIR-124A has diverse regulatory functions: it is upregulated during chondrogenesis [60], is a potential silencer of CDK6 when downregulated in leukemia [61], and regulates NFκB [62]. Except for MIR-124A, gene sets tend to have stronger enrichment in the greyhounds than in the Rottweilers. While this could suggest breed-specific OS pathways, it may also reflect greater tumor heterogeneity in the Rottweilers diluting the enrichment results.

## Discussion

Through a parallel multibreed canine association study, we found 33 genomic regions associated with OS, and identified genes and pathways potentially causing this complex, polygenic, and poorly understood disease. Altogether, the 33 loci identified by GWAS account for 50% to 80% of the disease risk within each of these three breeds, demonstrating that inherited factors are the predominant cause. In addition, regions of unusually low variability, reflective of the small effective population sizes and strong artificial selection used to create dog breeds, are also likely to contribute to an overall increased risk for these breeds.

None of the OS GWAS loci overlapped between breeds, a strikingly different genetic architecture from the shared variants previously found by mapping Mendelian traits in multiple dog breeds. Potentially this could reflect the difference between: (1) a monogenic trait, where a single variant causes a trait, which, if desirable, is then deliberately bred for; and (2) a complex trait, caused by a random assortment of many low frequency risk factors that rise in frequency through population bottlenecks and selection. This latter scenario is more similar to the genetics in human populations, where many rare risk factors may underlie OS, and may increase in frequency by random drift or moderate natural selection. In dog breeds, tighter bottlenecks and stronger selective forces push risk allele frequencies up, making them easier to detect. Thus, dogs bring added value to the study of OS.

The relatively large number of OS associated genes identified in this study facilitated pathway analysis with two different methods. First, the GRAIL software, given only the human genomic regions syntenic to the dog GWAS loci as input (and no information on phenotype) mined the abstracts of all previously published literature and found significant connections between associated genes related to growth, osteoblast differentiation and proliferation, and tumor suppression. Furthermore, a combined gene set enrichment analysis of the associated, fixed, and somatically altered loci identified common pathways affected by inherited and somatically acquired variation, suggesting they may interact to cause tumor initiation and progression. A fraction of the genes and pathways identified have previously been reported to be involved in human OS as either inherited or somatic changes, demonstrating the relevance of the canine model. Potentially more interesting, we also identified novel pathways, including two related to poorly characterized cis-regulatory motifs.

The GWAS loci implicated, several of which contain no known genes, show that canine OS has a complex genetic architecture with key advantages over artificially induced mouse models of the human disease. This is well illustrated by our functional analysis of the top greyhound locus. The strong effect of the variant and low genetic diversity of the breed populations allowed us to move rapidly from GWAS region to candidate functional variants with limited additional sequencing and genotyping. The top candidate causal variant is in a non-coding regulatory element upstream of the *CDKNA2A/B* locus, near, but not overlapping, a region associated with canine histiocytic sarcoma [63]. Pinpointing functional regulatory variants is harder than finding functional coding variation, but such regulatory variation is likely more representative of the genomic variants underlying common human diseases [64].

The *CDKNA2A/B* locus in dogs is syntenic to the human 9p21 locus, one of the most complex regulatory loci in the human genome [36]. SNPs in this region are significantly and independently associated with diseases including coronary artery disease, myocardial infarction, type 2 diabetes, melanoma, basal cell carcinoma, glioma, acute lymphoblastic leukemia, and breast cancer [64]. Deciphering the cellular mechanisms disrupted by OS associated regulatory variation in dogs may elucidate mechanisms underlying diverse human diseases.

We hypothesize that the top canine OS risk variant at chr11:44405676 alters regulation of *CDKN2A/ARF*. The OS associated variant at dog chr11:44405676 disrupts a highly constrained position in a genomic locus that we show, using a luciferase assay, has strong enhancer activity in a human osteosarcoma cell line. *CDKN2A/ARF* encodes the INK4 family of cyclin-dependent kinase inhibitor proteins (including p16^INK4a^, p15^INK4b^, and p14ARF). These proteins control G1-progression by inactivation of D-cyclins, inducing senescence via the RB and p53 pathways [65-67]. Altered levels of CDKN2A, a master regulator of tissue development, are linked to hematopoietic stem cell senescence and development, key feature of malignancies including OS [68,69]. SNPs that disrupt enhancer element binding can change transcriptional activity across the human 9p21 locus, including at *CDKN2A*[36]. Germline variants affecting regulation of *CDKN2A* may alter the balance between proliferation and senescence in specific tissues, thereby leading to an increased risk of developing OS and potentially also other cancers in adolescence and adulthood.

Canine and human OS are remarkably similar diseases, both clinically [8] and in their tumor gene expression profiles [6,7]. The recent publication of the first GWAS of osteosarcoma in humans offers a new opportunity to identify common risk factors shared between the two species and thus of particular etiological interest. The human OS GWAS compared 941 patients with osteosarcoma to 3,291 unaffected adults across 699,000 SNPs and found two genome-wide significant loci, one at the glutamate receptor gene *GRM4* and the other in a gene desert [5]. The much larger dataset required for GWAS in human patients illustrates the power offered by mapping in genetically isolated dog breed populations. While we see no association in the dog GWAS at the two loci found in the human GWAS, another glutamate receptor gene (*GRIK4*) is associated with OS in greyhounds. Although glutamate signaling in primary bone cancers is not well understood, both *GRM4* and *GRIK4* are expressed in normal bone, and glutamate signaling has been shown to regulate bone formation and resorption [70]. Furthermore, inhibition of glutamate receptors limits cell growth in many cell lines.

The association of glutamate receptors with OS in both dogs and humans suggests glutamate signaling as a potential therapeutic target, although the diverse physiological functions of this pathway could make this difficult. Glutamatergic signaling is critical for learning and memory [71] and is implicated in a range of neuropsychiatric diseases in both humans [72-74] and dogs [75]. Fixation of variants in glutamate related genes, as seen near GRM4 in Rottweilers (Additional file 1: Figure S9), may suggest that selection on behavioral, as well morphological, traits helped drive OS to the exceptionally high rates seen in the breeds studied here.

## Conclusions

Detailed characterization of the genetic risk factors involved in initiation and progression of canine OS will give us a comprehensive understanding of the genes involved and the different genetic interactions sufficient to cause the disease. We hypothesize that, by integrating genetic etiology into canine clinical trials, we can identify molecular subtypes of OS and correlate them with treatment efficacy and survival outcomes. In the future, we anticipate that the insights gained from the canine model will help us develop more personalized and effective treatments for human OS.

## Methods

### Sample collection and genotyping

DNA samples used were collected from pet dogs with the owner’s consent. Osteosarcoma was diagnosed by a qualified veterinarian using X-ray and/or tumor histopathology. Histological subtype was not included in GWAS analysis because of the difficulty of ascertaining it consistently across diverse collection sites. Osteosarcoma can be reliably diagnosed without histopathology in breeds with exceptionally high rates of the disease. Dogs classified as unaffected had no history of cancer. Phenotype included age at disease onset for most (79%) of affected dogs. For almost all (98%) of unaffected dogs, phenotype included age last confirmed OS free. Samples were collected with the appropriate consent and animal care protocols (U Minn 0802A27363, 1101A94713, and MIT 0910-074-13). Genomic DNA was isolated from whole blood (QIAamp DNA Midi kit) and was genotyped for approximately 170,000 SNPs using the Illumina 170 K canine HD array [17].

### Data analysis

SNPs and individuals with genotyping rate <0.95 were removed with PLINK [35]. Genetic relatedness and principal component (PC) analysis was done with GCTA [76]. To minimize population structure in the GWA analysis within each breed, we removed one dog from each concordant phenotype pair with genetic relatedness >0.25, preferentially retaining the younger case and older control. We measured phenotype-genotype association with the mixed model method implemented in EMMAX [77], which fits a standard linear regression model and tests the significance of the slope coefficient by the standard *t* test [77]. We included the top PC as a covariate to control for remaining cryptic relatedness [18].

We defined genome-wide significance in the GWAS using empirical 95% confidence intervals (CIs) rather than using a Bonferroni correction. As previously noted, a Bonferroni correction for the number of SNPs tested is excessively stringent in dog breeds, where extensive LD means that each SNP is not an independent test [14]. Of the approximately 100,000 SNPs used in the GWAS, the majority (98%) are in LD (r2 >0.5) with at least one other SNP within 1 Mb, and most (60%) are in LD with 10 or more SNPs. While an alternative is to correct for the number of independent haplotype blocks (if haplotype blocks are 1 Mb [14], the 2.4 Gb genome is comprised of roughly 2,400 independent haplotype blocks, corresponding to a corrected genome-wide significance threshold of 2 × 10^-5^), accurately determining the number of independent genomic regions in a particular breed population is difficult. We defined genome-wide significance using 95% CIs calculated from the empirical distribution of *P* values observed in the absence of real association. We determined this distribution by rerunning the GWAS with randomly permuted phenotypes 1,000 times. Conceptually, the empirical CIs bound the area in which 95% of the permutation QQ plots fall. We note that in dog breeds, the empirical CIs set more conservative thresholds than the CIs for a uniform score distribution used in, for example, human GWAS studies [78] (Additional file 1: Figure S10). We defined genome-wide significance as associations exceeding the 97.5% upper empirical CI, a threshold that varies by breed (1 × 10^-5^ in Greyhounds, 1 × 10^-4^ in Rottweilers, and 2 × 10^-4^ in IWH; Figure 2).

We defined genome-wide significant associations as those exceeding the 97.5% upper CI. To facilitate gene set and pathway analysis, we also identified a larger set of candidate OS regions with *P*<0.0005. The laxer threshold encompasses associations exceeding the theoretical CIs but not necessarily the stricter empirical CIs. We note that the inflated *P* values suggest that these regions are enriched for true OS associations. Although some of the regions included may not be true associations, this would most likely weaken rather than strengthen the gene set and pathway analyses, leading to false negatives rather than false positives.

We used linkage disequilibrium clumping in PLINK to define regions of association in two stages, first defining wide regions of SNPs in weak LD (r2 >0.2 within 5 Mb of top SNP) and then narrowing the association to a single peak of SNPs in strong LD (r2 >0.8 within 1 Mb of top SNP). While potentially masking a small number of true disease associated variants, this conservative approach ensured we identified only the strongest peaks and not regions with association reflecting proximity to a stronger peak. For each breed, we used GCTA to estimate the phenotype variance explained by the associated loci, which we defined broadly as SNPs with r^2^ >0.2 within 5 Mb of the peak SNP, totally 36.4 Mb for greyhound, 58.5 Mb for Rottweilers, and 18 Mb for IWH.

### Sequencing of top locus

We resequenced 5.9 Mb (chr11:43,000,000-48,900,000) in eight greyhound cases and seven controls using a Roche Nimblegen sequence capture array comprised of 385,000 probes covering approximately 95% of the target region. After library preparation (Illumina Paired end kit) DNA was hybridized to the array following the manufacturer’s protocol and the enriched samples sequenced using paired-end sequencing (2 × 74 bp) on the Illumina Genome analyzer II. Sequence reads were aligned to the CanFam2 genome reference using BWA [79]. Picard [80] was used to identify and mark duplicate reads (PCR artifacts) and the Genome analysis toolkit [81,82] to recalibrate quality scores, local realignment around indels, and call SNPs and indels. Variants were filtered based on sequence depth, quality of alignments, SNP clusters, and strand bias. In total, we identified 16,475 high quality variants; the Ts/Tv ratio was 2.06. The variants were annotated for cross-species conservation using SEQscoring [83,84], annotated and analyzed for predicted effect by using snpEff [85], and were visually examined by IGV [86].

We genotyped variants discovered in the sequence data using the Sequenom iPLEX Massarray system, tiling the center of the associated region (chr11:44.35 - 44.47 Mb) most densely. We genotyped 124 SNPs in greyhounds (172 A + 110 U) and Rottweilers (64 A + 32 U) and 60 SNPs in IWH (22 A + 30 U). SNPs were prioritized for genotyping if they were located in a protein coding sequence as defined by SnpEff [84] or in a conserved element compared to 29 other mammals [87]. We used Beagle 3.3.2 [88] to imputed genotype for all sequenced variants in LD with a genotyped variant (r2 >0.75) in the greyhounds and tested for association using PLINK [35]. We analyzed 41 bases around the top candidate SNP in greyhound for transcription factor binding motifs using FIMO [31] (JASPAR, UniPROBE, ENCODE-motifs databases, *P* value <1e-4) and TOMTOM [32] (JASPAR and UniPROBE databases, E-value <10) testing both the OS risk and non-risk alleles.

### Enhancer assay

For the enhancer assay, potential enhancer regions were PCR amplified from human gDNA and placed in front of minimal promoter driven luciferase reporter gene (pGL4.26, Promega). HTB-96 U-2 OS osteosarcoma cells were obtained from American Type Culture Collection (ATCC). These cells have been used within 6 months of purchase (February 2013). All cells were authenticated by standard ATCC procedures [89]. U-2 OS cells were seeded in 96 well plates (25,000 cells/well) and grown for 20 to 26 h before transfection. Each well was transfected with 0.1 μg reporter construct and 0.01 μg renilla luciferase driven by CMV promoter to control for cell density, using 0.4 μL/well FuGENE (Promega) according to the manufacturer’s instructions. Twenty-four hours after transfection, luciferase activity was measured sequentially using the Dual-Glo Luciferase System (Promega) using a Synergy H4 hybrid reader (BioTek). At least three independent experiments were performed, each with eight technical replicates of every construct.

### Pathway analysis

GRAIL analysis, using the PubMed Text (Aug2012) data source, was run on the OS regions (Table 1) lifted over to human genome hg18 coordinates (genome.ucsc.edu/cgi-bin/hgLiftOver) with 50 kb flanks added to start and end and gene size correction turned on. Gene set enrichment testing was done with INRICH, empirically measuring significance through 100,000 permutations matched for region size, SNP density, and gene number [50]. INRICH reports the significance for each gene set (*P*), and the experiment-wide significance correcting for the number of gene sets tested (*P*_corr_); thus, the correction varies depending on the number of sets included in the analysis. We considered *P*_corr_ <0.05 to be significant. We tested gene sets between 10 and 2,000 genes from five catalogs: three MSigDB collections (c2, c3, and c4 with 2593, 819, and 836 gene sets, respectively from [90]); the Gene Ontology ([91]; 1,582 sets); and the NCI/Nature pathway interaction database ([92]; 166 sets). We also made a custom catalog of putative osteosarcoma driver genes based on a recent publication [59]. We ran INRICH on canFam2 using a map file of 17,665 genes lifted over from the hg19 RefSeqGene catalog (UCSC Genome Brower) [93]. We made a custom catalog of 10 microRNA sets associated with OS in recent papers [51-55] and ran INRICH with a canFam2 map file of 532 human microRNAs mapped to dog with liftOver (Additional file 1: Table S6).

For each gene set we calculated the combined enrichment *P* value from the INRICH empirical *P* values for the GWAS and RRV regions using Fisher’s combined probability test. We first calculated the scores for each GWAS breed (that is, greyhound GWAS enrichment *P* combined with greyhound RRV enrichment *P*). We then determined the expected background distribution by doing the same using RRV enrichment test for each of a panel of 28 reference breeds, and then combining the GWAS enrichment *P* from each of the three GWAS breeds with the RRV enrichment *P* from each of the non-GWAS breeds [17].

### CGH

CGH analysis of primary canine OS was performed as described previously [94] using an approximate 180,000 feature Agilent canine oligonucleotide CGH array with approximately 26 kb resolution within the canFam 2.0 genome sequence assembly. An equimolar pool of constitutional DNA from 50 female dogs of mixed breed was used as the common reference for all OS cases. Arrays were scanned at 3 μm resolution using an Agilent G2565CA scanner and image data were processed using Feature Extraction version 10.10 and Genomic Workbench version 7.0 (Agilent Technologies) to exclude probes exhibiting non-uniform hybridization or signal saturation. Recurrent CNAs within each tumor were defined using the FASST2 segmentation algorithm in Nexus Copy Number version 6.1 (Biodiscovery Inc.) based on a minimum of three consecutive probes with log_2_ tumor:reference values ≥0.2 (copy number gain) or ≤ −0.2 (copy number loss). We compared genotypes at the top GWAS SNPs in Greyhound and Rottweiler (Table 1) to the CGH data from tumors for seven case greyhounds and seven case Rottweilers by first defining, for each probe, each dog’s phenotype (gain = log_2_ tumor:reference values ≥0.2 and loss = log_2_ tumor:reference values ≤0.2) and then associating phenotype with genotype using the Cochran-Mantel-Haenszel test in PLINK to control for breed clusters.

### Data availability

The data presented in this publication are available through the Gene Expression Omnibus (accession number GSE52147) and ArrayExpress (accession numbers E-MTAB-1984 and E-MTAB-1986). Datasets analyzed in the paper are also available at [95].

## Competing interests

The authors declare that they have no competing interests.

## Authors’ contributions

KLT, KC, and EKK conceived the study and together with JFM, MB, and MPS designed the experiment. KC, SS, GC, WK, MB, RT, JFM, MPS, NT, RS, SF, TB, NA MP, MS, CCC, LY, CAL, and SM coordinated, collected, prepared samples, made/confirmed diagnoses, and characterized samples for the study. SS, EI, MP, RS SF, TB, and NA performed genotyping, sequencing, and other genetic lab work. IE and JW performed the luciferase assays and other functional work. RT and MB generated and analyzed CGH data. EKK designed analysis methods. EKK, SS, EI, CH, SLR, ESL, and KLT performed and interpreted GWAS analysis, sequencing and association analysis, pathways analysis and risk assessment, motif prediction, and germline to somatic mutation analysis. EKK, SS, EI, and KLT wrote the paper with input from the other authors. All authors read and approved the final manuscript.

## Supplementary Material

Additional file 1: Figure S1 Regions of association defined with LD clumping. **Figure S2.** Ages of affected and unaffected samples. **Figure S3.** Phenotype variance explained at different association thresholds. **Figure S4.** The difference in genotype relative risk between cases and controls persists at more stringent significance thresholds. **Figure S5.** Cross-breed meta-analysis of OS GWAS datasets. **Figure S6.** Transcription factor motif analysis of top candidate variant. **Figure S7.** Size distribution of fixed and RRV regions by breed. **Figure S8.** CGH penetrance plots of dog and human OS. **Figure S9.** Dog GWAS results in human OS associated regions. **Figure S10.** Confidence interval comparison. **Table S1.** Association at top SNPs when sex is included as a covariate in the EMMAX genomewide analysis. **Table S2.** Top associated variants in the greyhound finemapping and imputation analysis. **Table S3.** Meta-analysis of nine breeds at the top candidate variant. **Table S4.** GRAIL keywords over-represented among genes in more than one osteosarcoma GWAS region. **Table S5.** Association between top GWAS SNPs and CGH gain/loss in matched germline tumor samples, controlling for breed clusters using Cochran-Mantel-Haenszel (CMH) test. **Table S6.** Osteosarcoma related microRNA sets curated from literature for INRICH testing. Click here for file
