# Supplementary material for: Genome-wide analyses implicate 33 loci in heritable dog osteosarcoma, including regulatory variants near CDKN2A/B
Source: Genome Biol. 2013 Dec 12;14(12):R132. doi: 10.1186/gb-2013-14-12-r132 (PMC4053774; doi:10.1186/gb-2013-14-12-r132)
Supplement: Additional file 1: Figure S1 — Regions of association defined with LD clumping. Figure S2. Ages of affected and unaffected samples. Figure S3. Phenotype variance explained at different association thresholds. Figure S4. The difference in genotype relative risk between cases and controls persists at more stringent significance thresholds. Figure S5. Cross-breed meta-analysis of OS GWAS datasets. Figure S6. Transcription factor motif analysis of top candidate variant. Figure S7. Size distribution of fixed and RRV regions by breed. Figure S8. CGH penetrance plots of dog and human OS. Figure S9. Dog GWAS results in human OS associated regions. Figure S10. Confidence interval comparison. Table S1. Association at top SNPs when sex is included as a covariate in the EMMAX genomewide analysis. Table S2. Top associated variants in the greyhound finemapping and imputation analysis. Table S3. Meta-analysis of nine breeds at the top candidate variant. Table S4. GRAIL keywords over-represented among genes in more than one osteosarcoma GWAS region. Table S5. Association between top GWAS SNPs and CGH gain/loss in matched germline tumor samples, controlling for breed clusters using Cochran-Mantel-Haenszel (CMH) test. Table S6. Osteosarcoma related microRNA sets curated from literature for INRICH testing. [file gb-2013-14-12-r132-S1.pdf]

Supplementary Information

Figure S1. Regions of association defined with LD clumping. For each of the 33 regions with $p < 0.0005$, we used a two stage approach to define the start and end (dashed lines) of the associated locus of SNPs in LD ($r^2 > 0.8$) to the top SNP in the region. For each SNP, the strength of LD to the top associated SNP is shown on a red (high) to blue (low) gradient.

Figure S2. Ages of affected and unaffected samples. For the majority of samples (91% of greyhounds, 74% of rottweilers and 93% of IWH) , we had the age of disease onset or the age last confirmed healthy. The IWH sample set had clear bimodal distribution with a higher proportion of young affected dogs, allowing us to compare young cases (< 6 years) to older controls (>6 years) while maintaining a sufficiently large sample set for GWAS analysis.

Figure S3. Phenotype variance explained at different association thresholds. Variance explained drops at more stringent association thresholds but is substantial even when just a handful of regions are included, suggesting a small number of strong loci underlie the high OS risk in these breeds. The phenotype variance explained (black line) and standard error (pink shaded area) are shown for each breed over a range of significance thresholds. The phenotype variance explained would be optimally estimated using just the causal loci, if known. Because of the small sample sizes and relatively small number of risk loci, estimating the variance using the whole genome gives large standard errors (0.32, 0.48 and 0.41 in the greyhounds, IWH and rottweilers respectively). Here, and in figure 2, we mitigated this effect by analyzing large genomic regions around significant association peaks. Average region size is 2.3Mb, 4.5Mb and 3.5Mb in the greyhounds, IWH and rottweilers. The number of regions included is shown along x axis.

Figure S4. The difference in genotype relative risk between cases and controls persists at more stringent significance thresholds. We estimated the relative risk for each dog based on the genotypes and odd ratios of SNPs at increasing significant thresholds (x axis). In each breed, the median GRR of affected dogs (red line) is distinct from the control dogs (black line) even at more stringent association thresholds than the 0.0005 used in figure 2. The number of SNPs included in the GRR is along the x axis. Shaded areas encompass the 25-75% percentiles of affected (pink) or unaffected (grey) dogs.

Figure S5. Cross breed meta-analysis of OS GWAS datasets. We combined the GWAS datasets in a random-effects meta-analysis using PLINK and found no evidence of an excess of high scoring SNPs when we compared the observed and expected p values for **a,** the meta-analysis of all three breeds, or (**b, c, d**) each possible pair of breeds. Dashed lines show 95% confidence intervals, with the red line indicated the median expected value.

Figure S6. Transcription factor motif analysis of top candidate variant. We tested both the wild-type and the risk allele for our top candidate variant using FIMO, and found 7 significant for the wild-type allele and 1 for the risk allele, using recommended significance thresholds. We tested all 8 with TOMTOM, and just one, for PAX5, was significantly detected by both tools and was specific to the non-risk allele (C, black box), suggesting that the risk allele (A) will disrupt binding.

Figure S7. Size distribution of fixed and RRV regions by breed. a, A substantial portion of the genome in each breed is fixed (SNPs with MAF < 0.05). The IWH breed has substantially more and longer blocks of fixation than the greyhounds or rottweilers. **b,** in each breed, we identified reduced relative variability (RRVs), the 1% of the genome that had exceptionally few segregating SNPs compared to 28 other breeds and includes regions of near, but not complete, fixation. Because the statistic accounts for the genomic background of the breed, the IWH are not longer outliers (Vaysse 2011).

Figure S8. CGH penetrance plots of dog and human OS. Array-based comparative genomic hybridization analysis (~26kb-resolution) demonstrates the extensive karyotypic instability of OS in **a,** greyhounds and **b,** rottweilers, with a large number of widely distributed CNAs (genomic gains in blue, losses in red) exceeding 50% penetrance. **c,** the CGH profiles of greyhound and rottweiler OS cases show remarkable global conservation. A two-tailed Fisher's Exact test of all CNAs with ≥20% differential penetrance identified regions with significantly different ($p<0.05$) copy number status between breeds (indicated by horizontal bars underneath the corresponding region in the comparison profile), but none are significant after multiple testing correction. **d,** recoding dog CGH data into human genomic coordinates highlights gross conservation with human OS (Angstadt et al. 2011), also evident at **e,** the top OS associated locus on near CDKN2A and CDKN2B on human chromosome 9.

Figure S9. Dog GWAS results in human OS associated regions. We see no association in dogs (red circles) at two loci significant in a human GWAS of osteosarcoma (dashed lines). Fixed SNPs (minor allele frequency < 0.05) are shown as black dots. Closer examination of SNP minor allele frequencies in the breeds (filled grey circles) shows extensive fixation in the IWH for the second GWAS locus on human chr 2, a gene desert, and shorter blocks of fixation in Rottweilers near the GRM4 locus on human chr 6.

Figure S10. Confidence interval comparison. We defined significant associations using empirically defined 95% confidence intervals (black lines) rather than the less conservative intervals expected for a uniform distribution (red dashed lines).

Table S1. Association at top SNPs when sex is included as a covariate in the EMMAX genomewide analysis.

Table S2. Top associated variants in the greyhound finemapping and imputation analysis.

Table S3. Meta analysis of 9 breeds at the top candidate variant

Table S4. GRAIL keywords overrepresented among genes in more than one osteosarcoma GWAS region

Table S5. Association between top GWAS SNPs and CGH gain/loss in matched germline tumor samples, controlling for breed clusters using Cochran-Mantel-Haenszel (CMH) test

Table S6. Osteosarcoma related microRNA sets curated from literature for INRICH testing.

Figure S1

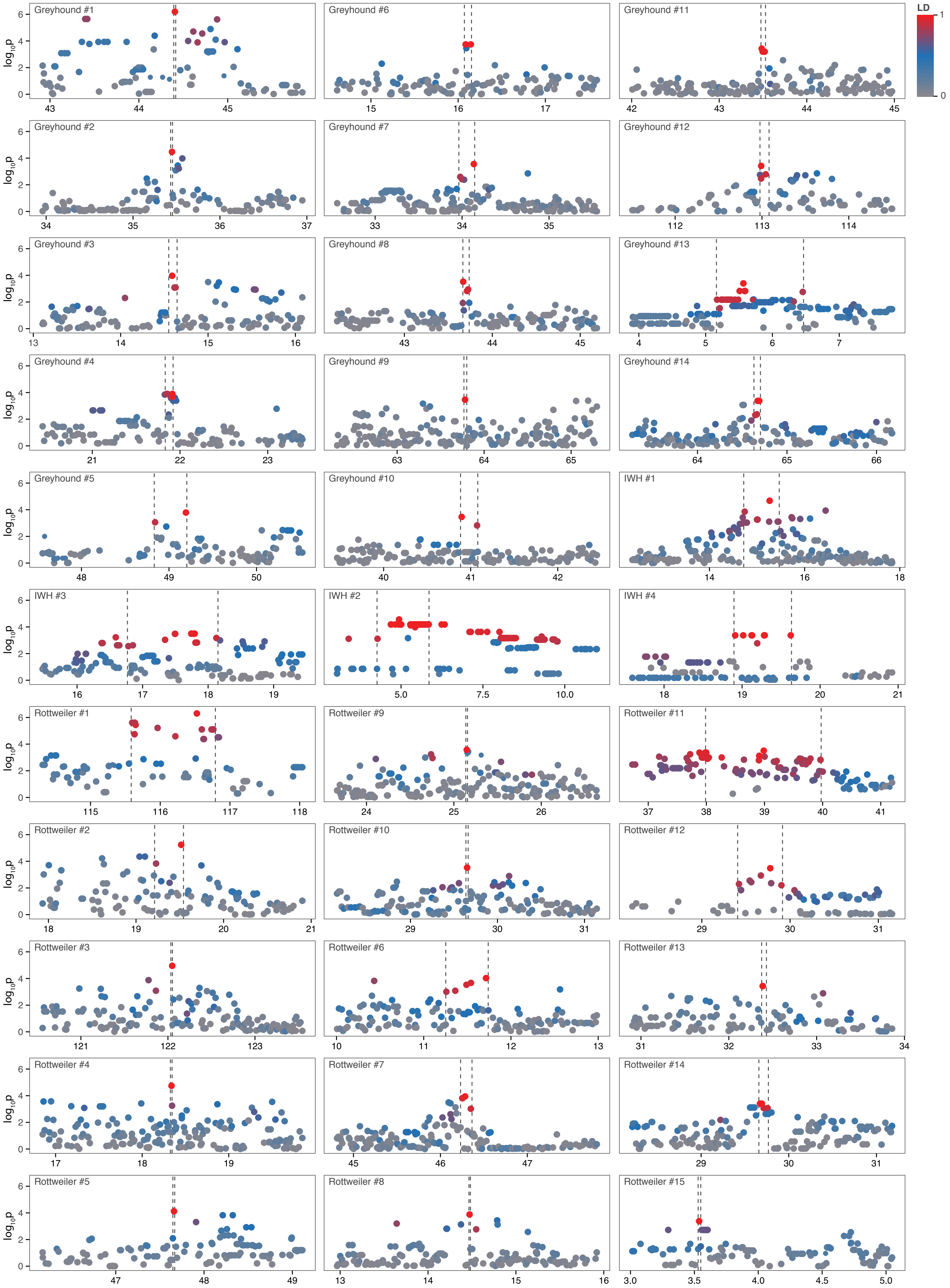

Greyhound #1
Greyhound #6
Greyhound #11
LD
1
0
log10p
Greyhound #2
Greyhound #7
Greyhound #12
Greyhound #3
Greyhound #8
Greyhound #13
Greyhound #4
Greyhound #9
Greyhound #14
Greyhound #5
Greyhound #10
IWH #1
IWH #3
IWH #2
IWH #4
Rottweiler #1
Rottweiler #9
Rottweiler #11
Rottweiler #2
Rottweiler #10
Rottweiler #12
Rottweiler #3
Rottweiler #6
Rottweiler #13
Rottweiler #4
Rottweiler #7
Rottweiler #14
Rottweiler #5
Rottweiler #8
Rottweiler #15

Figure S2

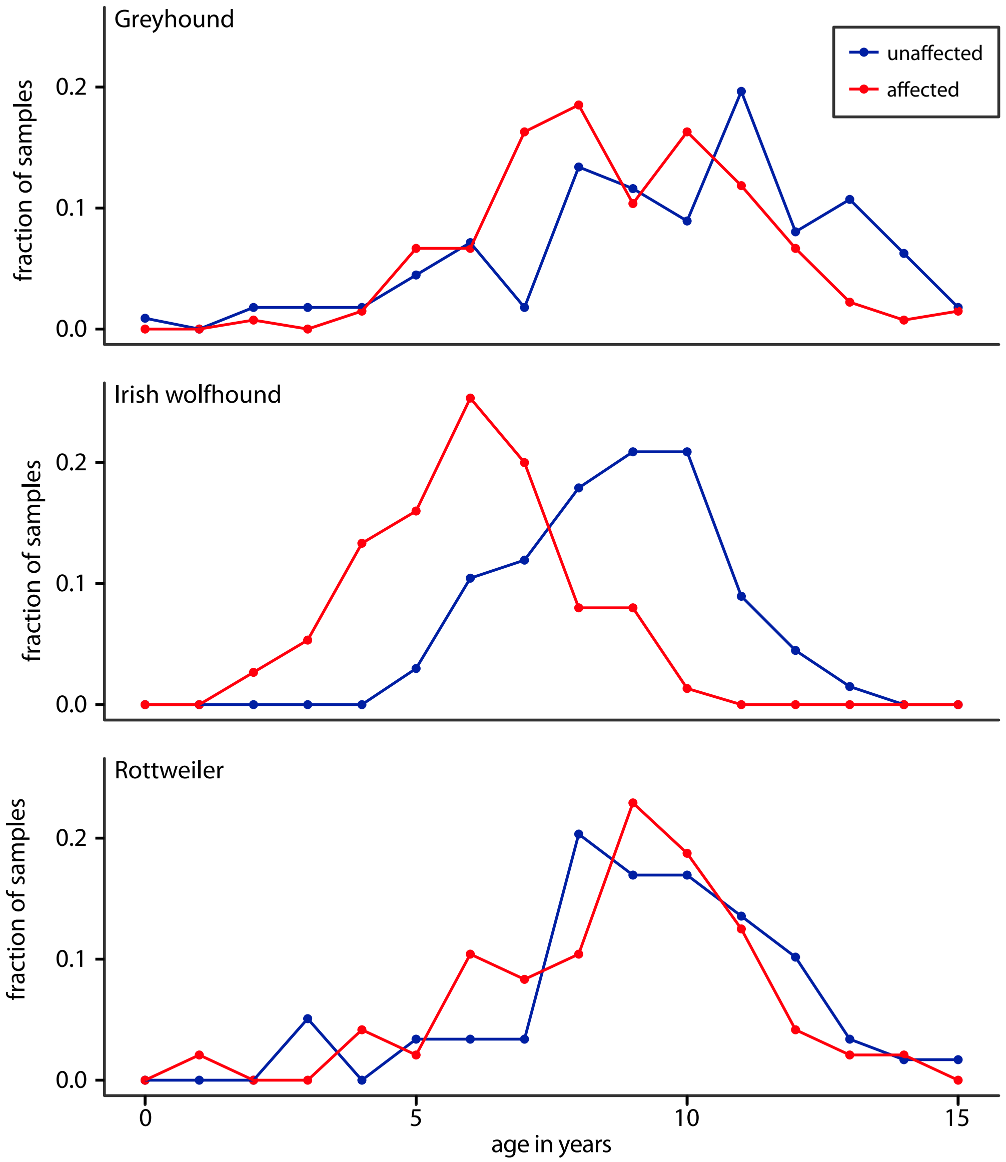
Greyhound
unaffected
affected
Irish wolfhound
Rottweiler
fraction of samples
0.0
0.1
0.2
0
5
10
15
age in years

Figure S3

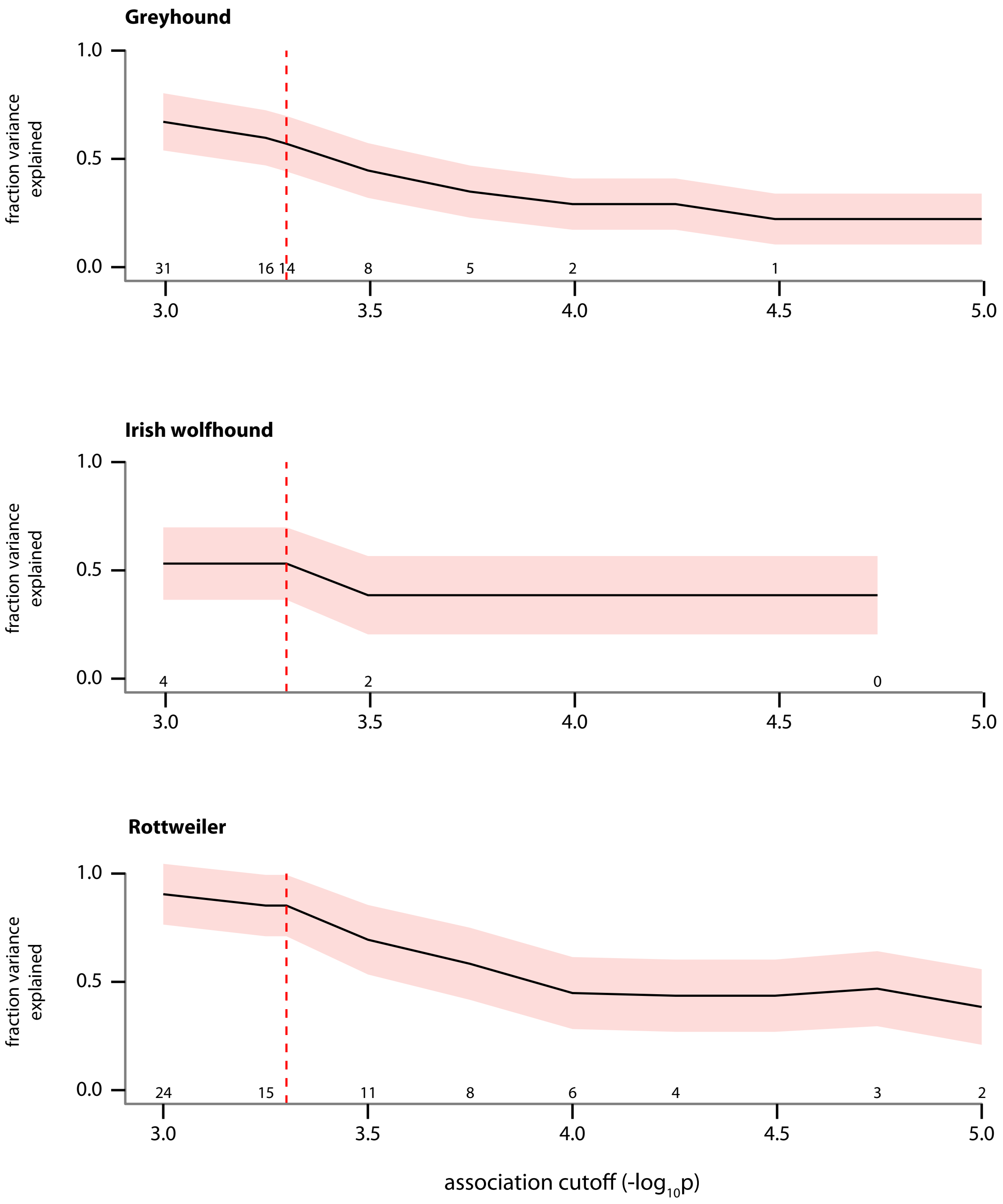
Greyhound
fraction variance explained
1.0
0.5
0.0
31
16
14
8
5
2
1
3.0
3.5
4.0
4.5
5.0
Irish wolfhound
fraction variance explained
1.0
0.5
0.0
4
2
0
3.0
3.5
4.0
4.5
5.0
Rottweiler
fraction variance explained
1.0
0.5
0.0
24
15
11
8
6
4
3
2
3.0
3.5
4.0
4.5
5.0
association cutoff (-log10p)

Figure S4

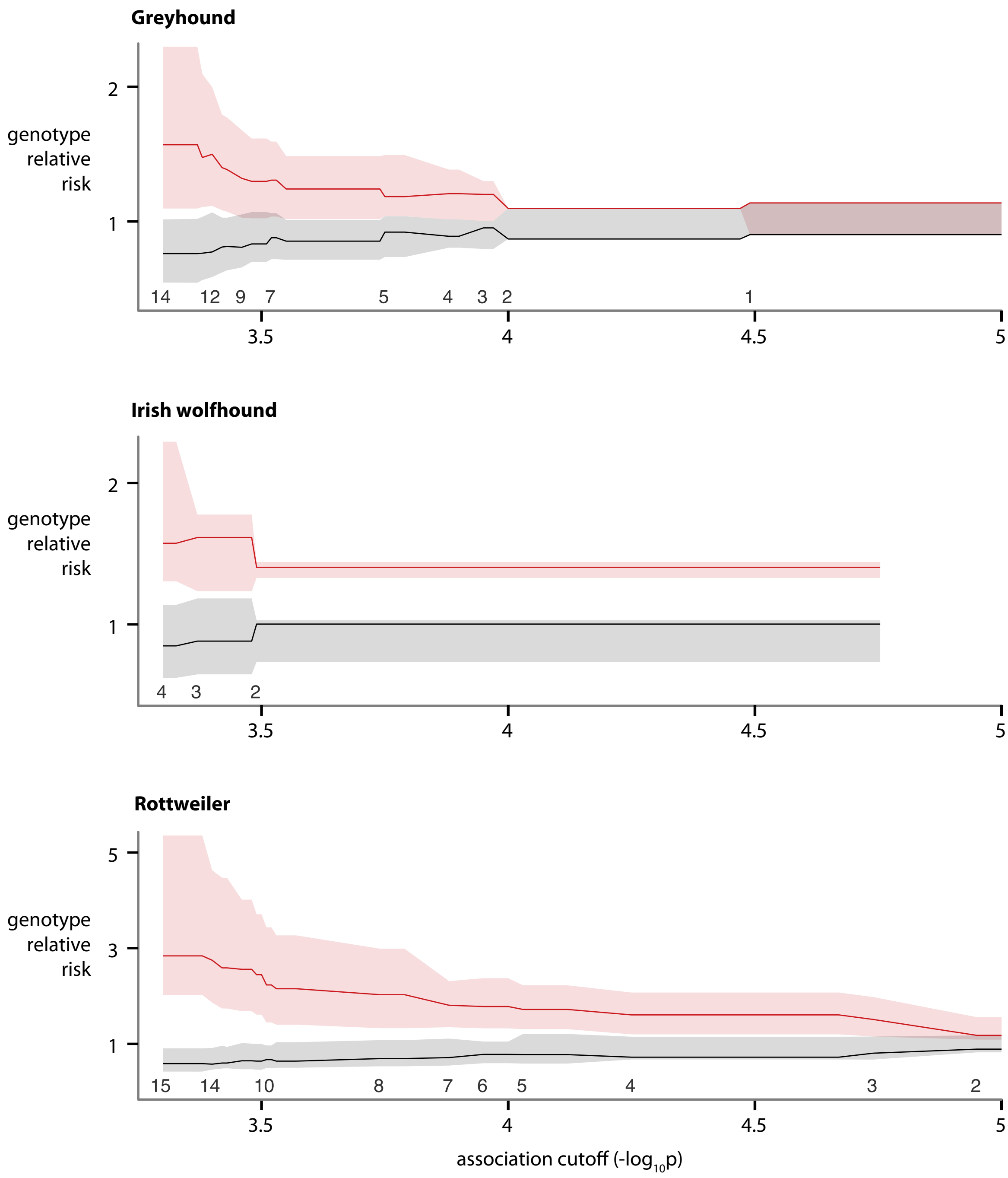

Greyhound
genotype relative risk
2
1
14
12
9
7
5
4
3
2
1
3.5
4
4.5
5
Irish wolfhound
genotype relative risk
2
1
4
3
2
3.5
4
4.5
5
Rottweiler
genotype relative risk
5
3
1
15
14
10
8
7
6
5
4
3
2
3.5
4
4.5
5
association cutoff (-log10p)

Figure S5

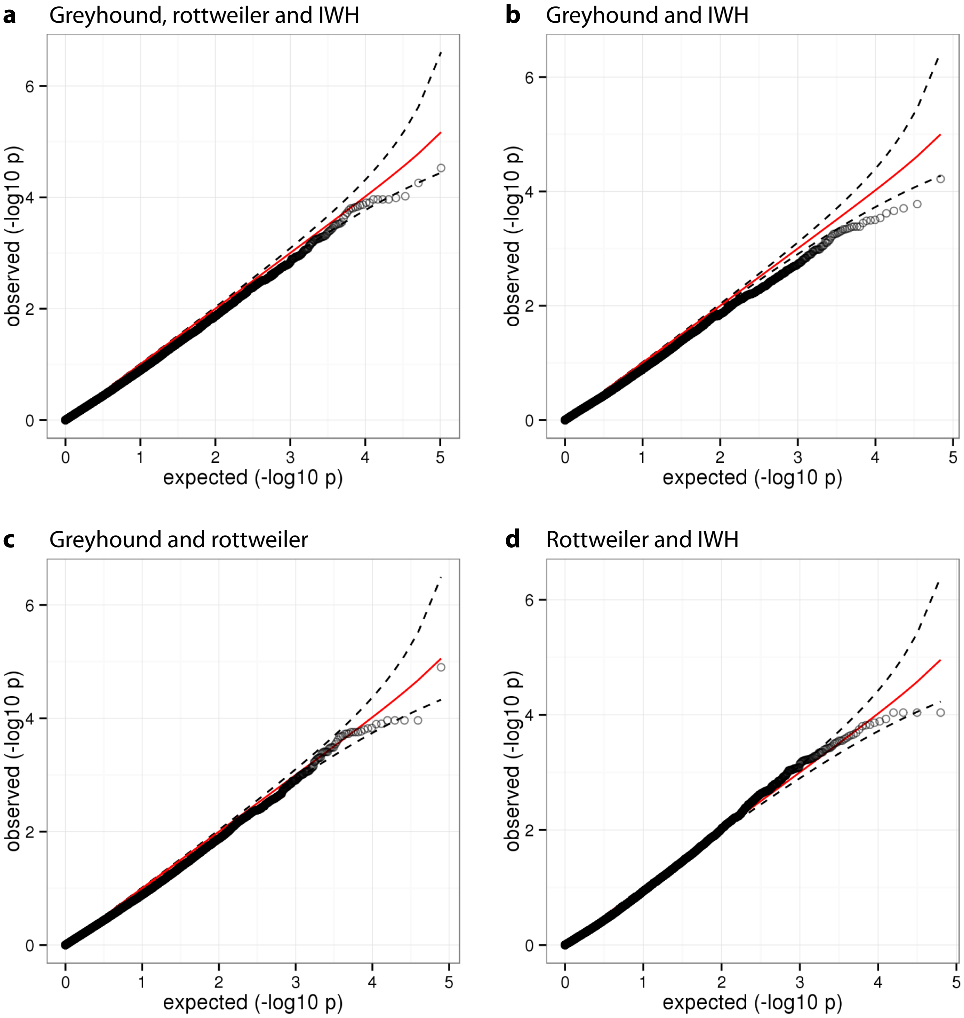
a
Greyhound, rottweiler and IWH
b
Greyhound and IWH
c
Greyhound and rottweiler
d
Rottweiler and IWH
observed (-log10 p)
expected (-log10 p)
0
2
4
6
0
1
2
3
4
5

Figure S6

	FIMO			TOMTOM
	$P_{reference}$	P_{risk}	*candidate variant*	$P_{reference}$
**T.F. motif**	**C allele**	**A allele**	CTGGCTGCTATG**C**GCCTTTGCTCCCGGG	**C allele**
CHD2_disc2	1.2E-05	*n.s.*		*n.s.*
E2F_disc5	1.2E-05	*n.s.*		*n.s.*
SP1_disc2	3.6E-05	*n.s.*		*n.s.*
Pax-5_known3	7.2E-05	*n.s.*		3.8E-03
RFX5_known4	9.3E-05	*n.s.*		*n.s.*
HNF4A/MA0114.1	*n.s.*	8.2E-05		1.1E-02
Rfxdc2_1/PB0056.1	4.5E-05	*n.s.*		3.0E-03
Rfx4_1/PB0055.1	6.4E-05	*n.s.*		7.8E-03

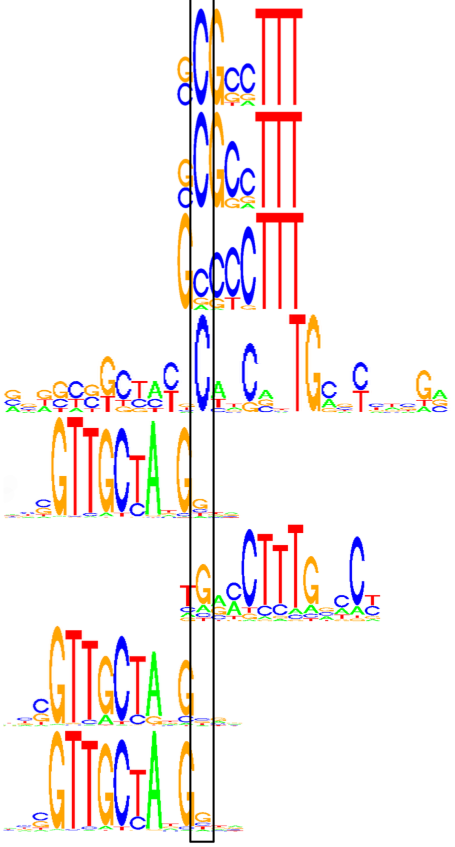

Figure S7

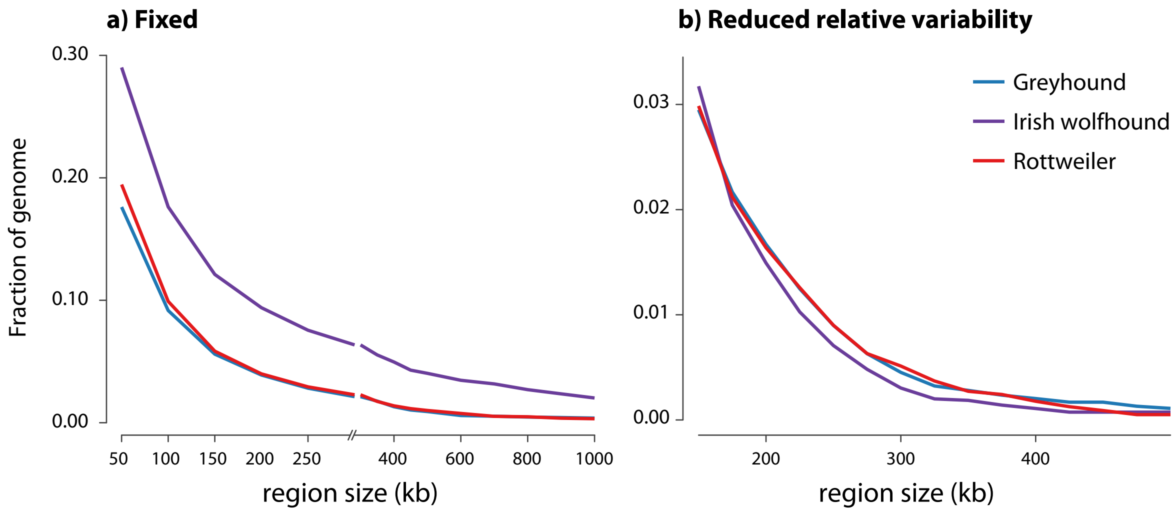
a) Fixed
0.30
0.20
0.10
0.00
Fraction of genome
50
100
150
200
250
400
600
800
1000
region size (kb)
b) Reduced relative variability
Greyhound
Irish wolfhound
Rottweiler
0.03
0.02
0.01
0.00
200
300
400
region size (kb)

Figure S8

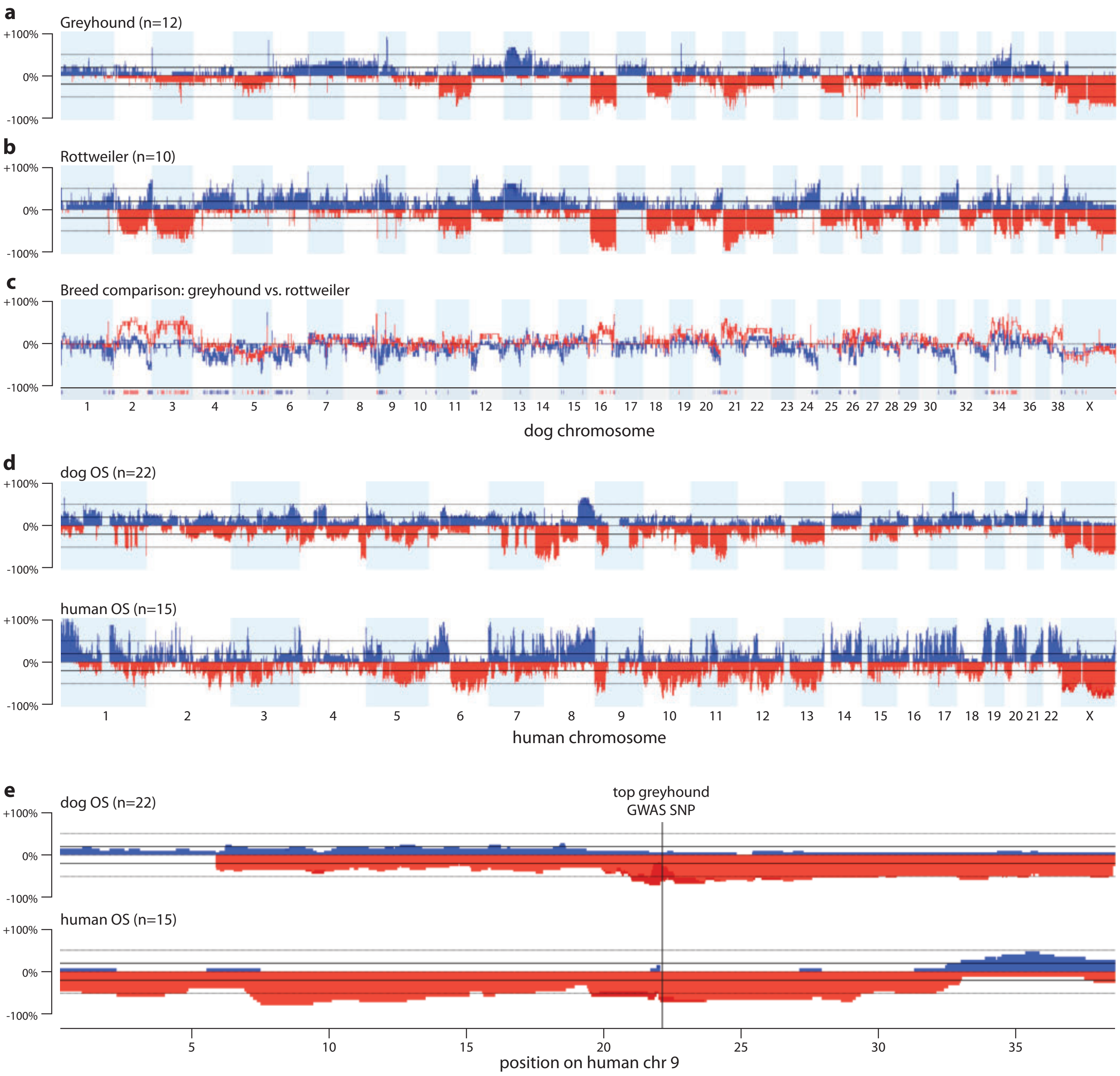
a
Greyhound (n=12)
+100%
0%
-100%
b
Rottweiler (n=10)
c
Breed comparison: greyhound vs. rottweiler
1 2 3 4 5 6 7 8 9 10 11 12 13 14 15 16 17 18 19 20 21 22 23 24 25 26 27 28 29 30 32 34 36 38 X
dog chromosome
d
dog OS (n=22)
human OS (n=15)
1 2 3 4 5 6 7 8 9 10 11 12 13 14 15 16 17 18 19 20 21 22 X
human chromosome
e
dog OS (n=22)
top greyhound
GWAS SNP
human OS (n=15)
5 10 15 20 25 30 35
position on human chr 9

Figure S9

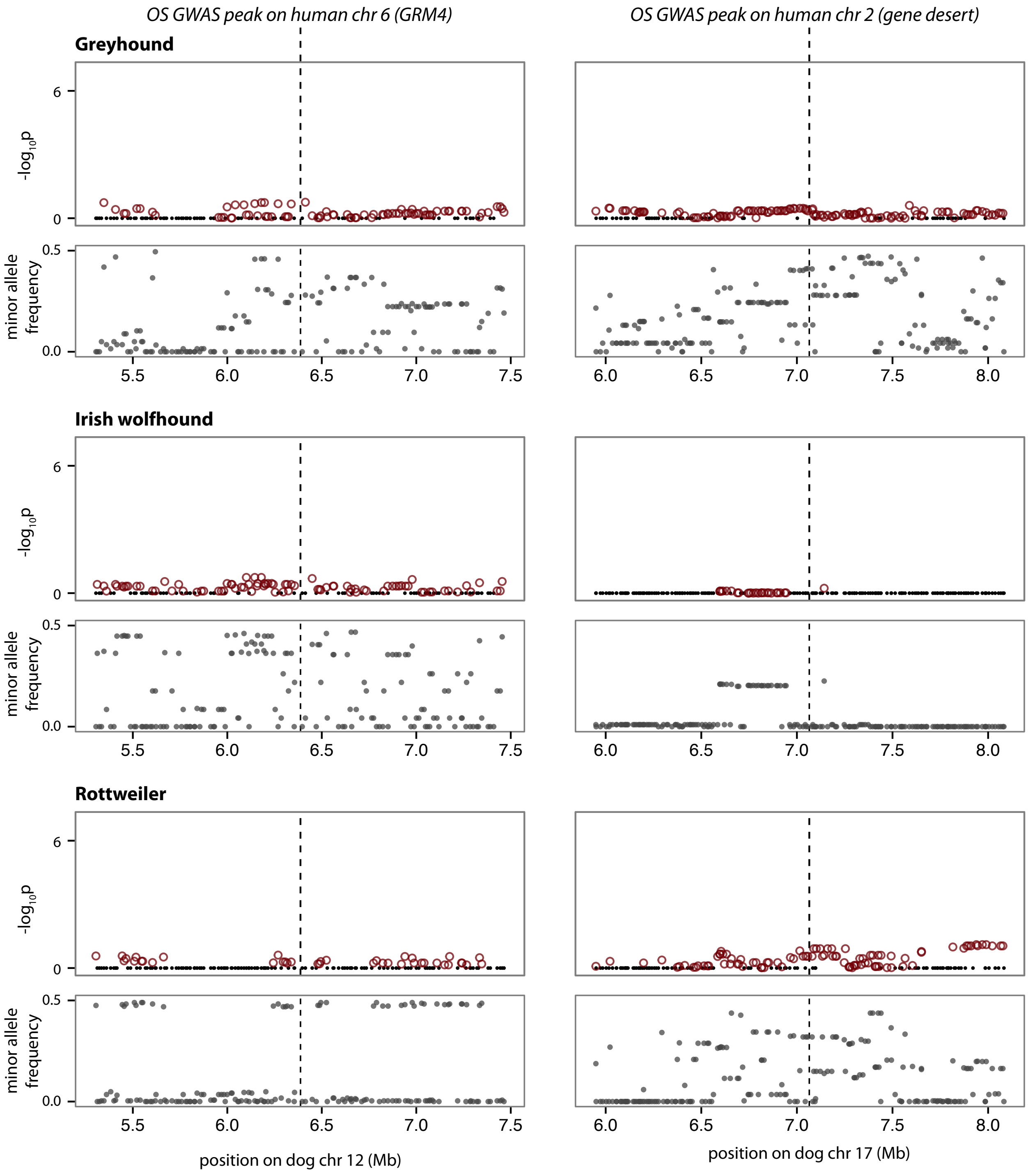

OS GWAS peak on human chr 6 (GRM4)
OS GWAS peak on human chr 2 (gene desert)
Greyhound
Irish wolfhound
Rottweiler
-log10p
minor allele frequency
6
0
0.5
0.0
5.5
6.0
6.5
7.0
7.5
8.0
position on dog chr 12 (Mb)
position on dog chr 17 (Mb)

Figure S10

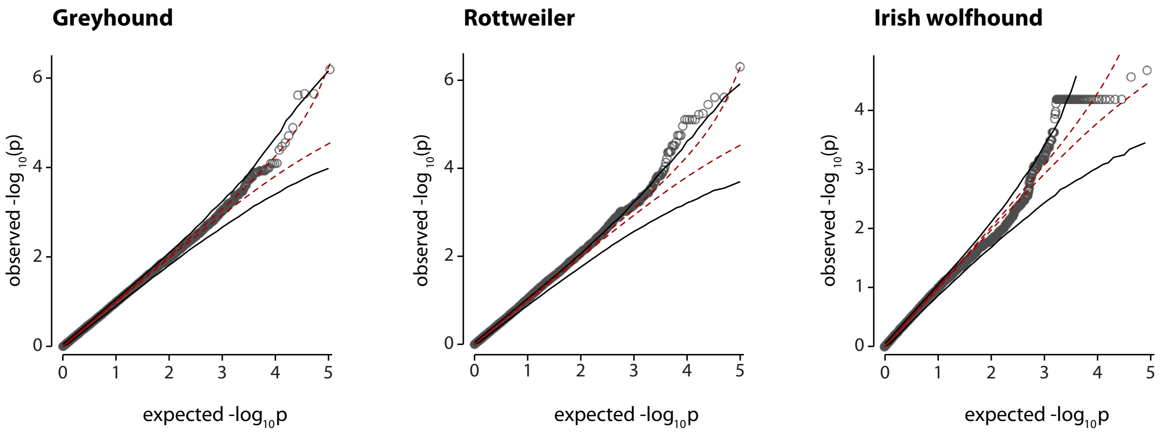

Greyhound
observed -log 10(p)
expected -log10p
Rottweiler
observed -log 10(p)
expected -log10p
Irish wolfhound
observed -log 10(p)
expected -log10p

Table S1. Association at top SNPs when sex is included as a covariate in the EMMAX genomewide analysis.

SNP and position	p (sex as covariate)	p (sex as phenotype)	f(A) males	f(U) males	f(A) females	f(U) females
Greyhound						
BICF2P133066 chr11:44405676	6.80E-07	0.86	0.84	0.66	0.85	0.63
BICF2P1421479 chr8:35448126	3.49E-05	0.13	0.15	0.02	0.09	0.03
BICF2S23118341 chr13:14588716	9.79E-05	0.23	0.31	0.18	0.39	0.2
BICF2S23325120 chr25:21912859	1.33E-04	0.43	0.56	0.44	0.55	0.37
BICF2P66597 chr14:49193217	1.63E-04	0.65	0.39	0.18	0.34	0.3
BICF2P1194727 chr5:16085937	1.89E-04	0.2	0.3	0.16	0.25	0.12
BICF2G63051809 chr19:34134931	2.87E-04	0.94	0.8	0.68	0.81	0.66
BICF2G630813090 chr16:43669044	3.14E-04	0.53	0.62	0.54	0.67	0.41
BICF2G630418573 chr15:63780452	3.55E-04	0.76	0.91	0.81	0.9	0.82
TIGRP2P215623 chr16:40896559	3.63E-04	0.38	0.97	0.9	0.96	0.88
TIGRP2P331221 chr25:43485109	3.87E-04	0.77	0.23	0.1	0.2	0.12
BICF2S23516022 chr1:112990983	4.01E-04	0.23	0.83	0.72	0.8	0.66
TIGRP2P45171 chr3:5564882	3.66E-04	0.13	0.76	0.68	0.84	0.68
BICF2P1090686 chr7:64672328	4.05E-04	0.36	0.54	0.42	0.6	0.44
Rottweiler						
BICF2P1115364 chr1:116524913	6.17E-07	0.4	0.7	0.35	0.73	0.43
BICF2P411325 chr2:19515571	6.23E-06	0.02	0.87	0.65	0.94	0.8
BICF2P1210630 chr1:122048812	1.38E-05	0.28	0.71	0.43	0.75	0.5
TIGRP2P407733 chr35:18338700	2.15E-05	0.28	0.5	0.22	0.52	0.36
BICF2P341331 chr9:47659782	8.98E-05	0.71	0.5	0.31	0.56	0.25
BICF2P1129874 chr38:11714169	8.49E-05	0.62	0.5	0.3	0.49	0.23
TIGRP2P286750 chr21:46283811	7.28E-05	0.13	0.86	0.78	0.83	0.63
BICF2S23533459 chr17:14472761	1.51E-04	0.63	0.17	0	0.18	0.02
BICF2G630590368 chr32:25147661	2.80E-04	0.59	0.92	0.81	0.98	0.8
BICF2P92014 chr36:29651125	3.27E-04	0.92	0.68	0.41	0.63	0.43
TIGRP2P200071 chr15:38987072	2.22E-04	0.1	0.91	0.59	0.74	0.59
BICF2P1164085 chr1:29775073	3.37E-04	0.99	0.47	0.2	0.44	0.25
BICF2S23712115 chr26:32385934	3.65E-04	0.96	0.92	0.74	0.89	0.79
BICF2G63095567 chr25:29671618	3.99E-04	0.92	0.26	0.02	0.19	0.09
BICF2P841536 chr26:3537143	4.62E-04	0.34	0.42	0.31	0.52	0.29
Irish Wolfhound						
BICF2S23746532 chr5:15264066	1.01E-05	0.48	0.67	0.17	0.34	0.16
BICF2P1466354 chr18:4937944	7.01E-05	0.26	0.39	0.2	0.64	0.21
BICF2P1225386 chr1:17742179	1.54E-04	0.49	0.67	0.24	0.37	0.16
BICF2P1125643 chr9:19623231	6.57E-04	0.72	0.11	0.02	0.16	0.01

Table S2. Top associated variants in the greyhound finemapping and imputation analysis

Position on dog chr 11	Frag-ment*	variant type	typing method	human coordinate	p	risk allele	f (case)	f (control)	non-risk allele	Can-Fam2 allele	hg19
44390632	A,B	SNP	geno-typed	chr9 22130885	9.1E-08	T	0.86	0.68	C	C	C
44391818	C	SNP	geno-typed	chr9 22132592	9.1E-02	A	0.95	0.91	C	A	A
44392971	C	SNP	geno-typed	chr9 22133979	1.8E-07	G	0.92	0.77	A	G	G
44397317		INDEL	imputed	nf	3.2E-08	C	0.87	0.68	CG	C	*nf*
44401361-44401371	F	DEL & SNP	imputed	chr9 22141194-22141211	3.2E-08	G*	0.87	0.68	A**	A	A
44402703	F	SNP	geno-typed	chr9 22142611-22142618	1.3E-05	C	0.97	0.87	T	C	*nf*
44405676	G	SNP	imputed & validated	chr9 22148443	3.2E-08	A	0.87	0.68	C	C	C

* luciferase assay fragment covering region (figure 3C)
**variant site consists of a combined 8 bp deletion and a SNP
nf = matched region not found in human genome build hg19

Table S3: Meta analysis of 9 breeds at top candidate variant

breed	# aff	# unaff	risk allele*	Frisk (aff)	F (unaff)	P (controlling for breed)	odds ratio with 95% C.I.	$p_{Breslow-Day}$ (OR hetero-geneity)
Greyhound	179	115	**A**	0.87	0.68	4.3E-08	3.06 (2.03-4.62)	
Leonberger	30	25	**A**	0.77	0.62	0.095	2.01 (0.88-4.61)	
Great Pyrenees	16	21	**A**	0.78	0.62	0.135	2.2 (0.77-6.24)	
Irish Wolfhound	27	31	**A**	0.93	0.92	0.895	1.1 (0.28-4.31)	
Rottweiler	92	77	**A**	0.98	0.97	0.542	1.51 (0.4-5.72)	
Mastiff	14	13	*C*	0.32	0.31	0.91	1.07 (0.34-3.37)	
Golden retreiver	37	36	*C*	0.95	0.92	0.48	1.59 (0.43-5.89)	
Labrador retriever	10	20	*C*	0.30	0.15	0.17	2.43 (0.67-8.84)	
Great Dane	26	24	*C*	0.25	0.10	0.06	2.87 (0.94-8.78)	
Risk breeds (greyhound, Leonberger, Great Pyrenees, Irish wolfhound, rottweiler)	383	300	**A**			1.7E-08	2.5 (1.81-3.46)	0.51
Risk breeds without greyhound (Leonberger, Great Pyrenees, Irish wolfhound, rottweiler)	202	185	**A**			0.032	1.79 (1.05-3.06)	0.85
All 9 breeds	487	420	**A**			7.9E-05	1.73 (1.31-2.28)	1.1E-03
All breeds except greyhound (8 breeds)	306	305	**A**			0.820	1.05 (0.71-1.54)	0.12

* risk allele is defined as allele with higher frequency in cases than in controls

Table S4. GRAIL keywords overrepresented among genes in more than one osteosarcoma GWAS region

GWAS region	Gene	GRAIL p	#1 "bone"	#2 "rank"	#3 "bleomycin"	#4 "rankl"	#5 "neural"	#6 "differentiation"	#7 "development"	#8 "morphogenetic"	#9 "locus"	#10 "endothelial"	#11 "engrailed"	#12 "coronary"	#13 "notch"	#14 "snps"	#15 "patterning"	#16 "embryos"	#17 "artery"	#18 "signaling"
**Greyhound**																				
2	OTX2	0.0014	X					X		X	X				X					X
5	BMPER	0.0014	X				X	X	X		X				X		X	X		
7	EN1	0.0047	X			X	X	X	X	X	X				X					X
11	CCL20	0.1994	X			X		X				X							X	
12	FOSB	0.0836	X				X	X		X									X	
	ERCC2	0.1244		X	X											X				
**Irish wolfhound**																				
1	BLID	0.0198									X					X				
2	VWC2	0.0007	X						X									X		X
	IKZF1	0.0402	X					X	X	X	X				X		X	X	X	X
	DDC	0.1615					X	X	X	X	X		X		X	X		X		
3	TNFRSF11A	0.0093						X	X	X	X					X				X
	BCL2	0.0240	X	X	X		X	X	X			X		X				X	X	X
**Rottweiler**																				
1	DLL3	0.0009					X	X	X	X	X		X	X			X	X		
	MIA	0.0165	X					X		X										
	LGALS14	0.0615												X		X				
	NUMBL	0.0657	X				X	X	X		X							X		X
	EID2	0.1459																		X
	CYP2A6	0.1582														X				
	ITPKC	0.1585									X			X		X			X	
	AKT2	0.1586	X	X		X		X	X	X		X		X		X			X	X
	PRX	0.1589									X									X
	C19orf47	0.1648							X									X		
2	KIAA1462	0.0057										X								
5	BLMH	0.1126						X			X									
6	FAM5C	0.0470	X				X	X		X	X			X		X			X	
7	NELL1	0.0008					X		X	X	X					X	X			X
9	EMCN	0.0074	X				X	X	X					X	X		X	X	X	X
11	LTA4H	0.0766	X									X		X		X			X	
	NTN4	0.1896					X		X	X		X							X	
12	TCF21	0.0042	X				X	X		X	X	X	X	X	X		X	X	X	X

Table S5. Association between top GWAS SNPs and CGH gain/loss in matched germline tumor samples, controlling for breed clusters using Cochran-Mantel-Haenszel (CMH) test

SNP	risk allele	CGH probe	P (CMH)	genes	pheno-type	breed	# affect	# unaff	f (aff)	f (unaff)	p_{breed}
chr9 47659782 (rottweiler region 5)	A	chr9 47652378-47652437	0.008	BLMH	gain	grey	5	2	0.40	0.00	0.135
						rott	5	2	0.70	0.00	0.018
chr1 112990983 (greyhound region 12)	C	chr1 112987331-112987390	0.020		gain	grey	5	2	1.00	0.50	0.016
						rott	2	5	1.00	1.00	*fixed*
chr1 29775073 (rottweiler region 12)	G	chr1 29783871-29783930	0.020	ERCC1, FOSB	gain	grey	0	7		0.50	
						rott	1	6	1.00	0.17	0.016
chr9 47659782 (rottweiler region 5)	A	chr9 47663854-47663913	0.023	TMIGD1	loss	grey	0	7		0.29	
						rott	2	5	0.00	0.70	0.018
chr2 19515571 (rottweiler region 2)	C	chr2 19524472-19524531	0.043		loss	grey	1	6	0.50	0.00	0.011
						rott	1	6	1.00	0.92	0.672

Table S6. Osteosarcoma related microRNA sets curated from literature for INRICH testing.

Source	# genes	genes (autosomal genes mapped to canFam2)	genes in 3 breed fixed regions*	INRICH p	INRICH $p_{corrected}$
Jones 2012	28	MIR10B, MIR126, MIR142, MIR150, MIR15B, MIR16-2, MIR181C, MIR190, MIR190A, MIR195, MIR210, MIR214, MIR26B, MIR27A, MIR326, MIR335, MIR340, MIR451, MIR483, MIR486, MIR487A, MIR488, MIR574, MIR616, MIR650, MIR657, MIR663, MIRLET7G	MIR150, MIR335, MIR340, MIR650, MIR663	**0.017**	**0.041**
Lulla 2011	15	MIR126, MIR135B, MIR140, MIR142, MIR148A, MIR150, MIR18A, MIR198, MIR200B, MIR210, MIR301B, MIR451, MIR454, MIR455, MIR511-1	MIR140, MIR150	0.032	0.073
Maire 2011	33	MIR100, MIR126, MIR127, MIR137, MIR142, MIR148A, MIR154, MIR181A1, MIR181A2, MIR195, MIR199B, MIR218-1, MIR218-2, MIR299, MIR31, MIR329-1, MIR329-2, MIR335, MIR376A1, MIR376A2, MIR376C, MIR377, MIR382, MIR409, MIR410, MIR432, MIR451, MIR493, MIR495, MIR497, MIR543, MIR654, MIR758	MIR335	0.491	0.814
Sarver 2010	21	MIR130B, MIR144, MIR154, MIR17, MIR18A, MIR20A, MIR369, MIR377, MIR381, MIR409, MIR431, MIR449A, MIR493, MIR539, MIR614, MIR642, MIR642A, MIR653, MIR668, MIRLET7C, MIRLET7E		1.000	1.000
Thayanithy 2012	28	MIR106B, MIR107, MIR125B1, MIR130B, MIR134, MIR149, MIR150, MIR15A, MIR17, MIR198, MIR19A, MIR32, MIR33A, MIR33B, MIR345, MIR369, MIR370, MIR382, MIR409, MIR425, MIR432, MIR433, MIR449A, MIR487A, MIR511-1, MIR539, MIR544, MIR645	MIR150	0.337	0.627
Thayanithy 2012 (downregulated 2x)	14	MIR125B1, MIR150, MIR198, MIR369, MIR370, MIR376A1, MIR382, MIR409, MIR432, MIR433, MIR487A, MIR511-1, MIR539, MIR645	MIR150	0.195	0.437
Thayanithy 2012 (upregulated 2x)	14	MIR106B, MIR107, MIR130B, MIR149, MIR15A, MIR17, MIR19A, MIR219-1, MIR32, MIR33A, MIR33B, MIR345, MIR425, MIR449A		1.000	1.000
all OS microRNAs	107	MIR100, MIR106B, MIR107, MIR10B, MIR125B1, MIR126, MIR127, MIR130B, MIR132, MIR134, MIR135B, MIR137, MIR140, MIR142, MIR143, MIR144, MIR148A, MIR149, MIR150, MIR151, MIR154, MIR15A, MIR15B, MIR16-2, MIR17, MIR181A1, MIR181A2, MIR181C, MIR18A, MIR190, MIR190A, MIR191, MIR193A, MIR195, MIR198, MIR199A1, MIR199B, MIR19A, MIR200B, MIR20A, MIR21, MIR210, MIR214, MIR218-1, MIR218-2, MIR219-1, MIR26B, MIR27A, MIR299, MIR301B, MIR31, MIR32, MIR326, MIR329-1, MIR329-2, MIR335, MIR33A, MIR33B, MIR340, MIR345, MIR369, MIR370, MIR376A1, MIR376A2, MIR376C, MIR377, MIR381, MIR382, MIR409, MIR410, MIR422A, MIR425, MIR431, MIR432, MIR433, MIR449A, MIR451, MIR454, MIR455, MIR483, MIR486, MIR487A, MIR488, MIR493, MIR495, MIR497, MIR511-1, MIR539, MIR543, MIR544, MIR574, MIR614, MIR616, MIR642, MIR642A, MIR645, MIR650, MIR653, MIR654, MIR657, MIR663, MIR668, MIR758, MIR99B, MIRLET7C, MIRLET7E, MIRLET7G	MIR140, MIR150, MIR335, MIR340, MIR650, MIR663	0.025	0.052

** regions of overlap of fixed regions (regions > 250kb where all SNPs have MAF < 0.05) from greyhounds, rottweilers and IWH*
